# NLRP6 controls pulmonary inflammation from cigarette smoke in a gut microbiota-dependent manner

**DOI:** 10.3389/fimmu.2023.1224383

**Published:** 2023-12-11

**Authors:** Mégane Nascimento, Sarah Huot-Marchand, Manoussa Fanny, Marjolène Straube, Marc Le Bert, Florence Savigny, Lionel Apetoh, Jacques Van Snick, Fabrice Trovero, Mathias Chamaillard, Valérie F. J. Quesniaux, Bernhard Ryffel, Philippe Gosset, Aurélie Gombault, Nicolas Riteau, Harry Sokol, Isabelle Couillin

**Affiliations:** ^1^University of Orleans and Centre National de Recherche scientifique (CNRS), Experimental and Molecular Immunology and Neurogenetics (INEM)-UMR7355, Orleans, France; ^2^Sorbonne Université, Institut National de la Recherche Médicale (INSERM), Centre de Recherche Saint-Antoine (CRSA), Assistance Publique-Hôpitaux de Paris (AP-HP), Hôpital Saint Antoine, Service de Gastroenterologie, Paris, France; ^3^INSERM, U1100, Tours, France; ^4^Ludwig Cancer Research, Brussels, Belgium; ^5^ArtImmune SAS, Orleans, France; ^6^Univ. Lille, Institut National de la Recherche Médicale (INSERM), U1003 - Laboratoire de physiologie cellulaire (PHYCEL) - Physiologie Cellulaire, Lille, France; ^7^Institut PASTEUR INSERM U1019, Centre National de Recherche (CNRS) Unité Mixte de Recherche (UMR) 8204, Lille, France; ^8^Institut national de la recherche agronomique (INRA), UMR1319 Micalis, AgroParisTech, Jouy-en-Josas, France; ^9^Paris Centre for Microbiome Medicine (PaCeMM) FHU, Paris, France

**Keywords:** NLRP6, lung inflammation, cigarette smoke-exposure, gut microbiota, gut to lung axis

## Abstract

Chronic obstructive pulmonary disease (COPD) is a major health issue primarily caused by cigarette smoke (CS) and characterized by breathlessness and repeated airway inflammation. NLRP6 is a cytosolic innate receptor controlling intestinal inflammation and orchestrating the colonic host–microbial interface. However, its roles in the lungs remain largely unexplored. Using CS exposure models, our data show that airway inflammation is strongly impaired in Nlrp6-deficient mice with drastically fewer recruited neutrophils, a key cell subset in inflammation and COPD. We found that NLRP6 expression in lung epithelial cells is important to control airway and lung tissue inflammation in an inflammasome-dependent manner. Since gut-derived metabolites regulate NLRP6 inflammasome activation in intestinal epithelial cells, we investigated the link between NLRP6, CS-driven lung inflammation, and gut microbiota composition. We report that acute CS exposure alters gut microbiota in both wild-type (WT) and Nlrp6-deficient mice and that antibiotic treatment decreases CS-induced lung inflammation. In addition, gut microbiota transfer from dysbiotic Nlrp6-deficient mice to WT mice decreased airway lung inflammation in WT mice, highlighting an NLRP6-dependent gut-to-lung axis controlling pulmonary inflammation.

## Graphical Abstract

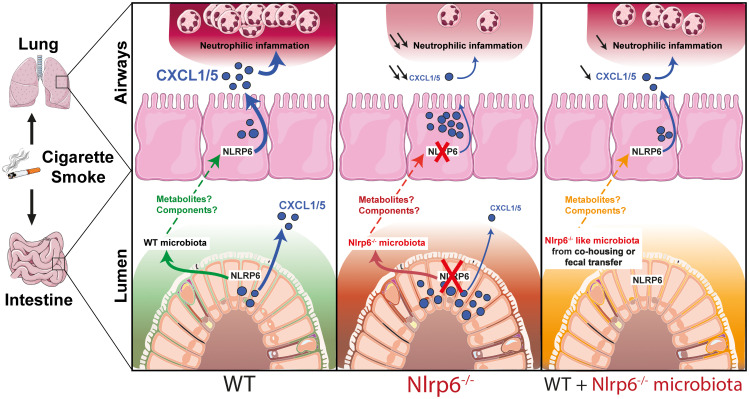

## Highlights

- NLRP6 controls lung inflammation from cigarette smoke through CXCL1/CXCL5-mediated neutrophilic influx
- NLRP6-mediated lung inflammation is dependent on the inflammasome
- NLRP6 expression in airway epithelial cells controls lung inflammation from cigarette smoke
- Cigarette smoke exposure alters gut microbiota
- Impaired lung inflammation in cigarette smoke-exposed Nlrp6-deficient mice is transferable to wild-type mice by co-housing experiments or fecal microbiota transplantation
- Gut microbiota from Nlrp6-deficient mice limit neutrophilic lung inflammation

## Introduction

Chronic obstructive pulmonary disease (COPD) is a severe inflammatory disease characterized by airway obstruction due to bronchial immune cell recruitment and mucus secretion, resulting in impaired lung function. In a fraction of patients, chronic bronchial inflammation evolves into alveolar disease with alveolar wall destruction, leading to enlargement of the airway, which causes shortness of breath and is known as emphysema (1). COPD is a major health issue affecting 4 to 10% of the worldwide population and is the third cause of death in the world. If appropriate action is not taken, 600 million people will suffer from COPD in 2050 (2). Cigarette smoking is a major societal cause of COPD, accounting for more than 95% of cases in industrialized countries (3, 4). Inflammation can persist even when smoking ceases. Current therapeutics target symptoms, attempting to alleviate chronic inflammation and prevent infection-driven exacerbations; however, there is no disease-modifying treatment available (5). Chronic inflammation is characterized by the recruitment and activation of innate immune cells, in particular neutrophils and macrophages as well as adaptive immune cells such as lymphocytes, which produce inflammatory and tissue damaging mediators (6–8). Inflammatory neutrophils display increased degranulation and reactive oxygen species release and have been associated with COPD severity (9). Neutrophils target lung tissue through ELR^+^ CXC chemokines (i.e. containing a tripeptide motif glutamic acid-leucine-arginine and one amino acid between the first two cysteines), essential for both neutrophil influx and activation (10) through signaling on the transmembrane receptors CXCR1 and CXCR2. In particular, CXCL5/LIX (LPS-induced CXC chemokine) is mainly produced by epithelial cells and promotes airway neutrophil inflammation (11–15). In contrast, alveolar macrophages secrete CXCL1/KC and CXCL2/MIP-2 but not CXCL5/LIX (11). The important contributions of epithelial cell-derived chemokines to immunity have recently been highlighted (16, 17).

Using mouse models of cigarette smoke (CS) exposure and elastase-induced injury, we previously showed that interleukin (IL-) 1β and the adaptor molecule apoptosis-associated speck-like protein containing a CARD (ASC) are essential to promote inflammation, remodeling, and emphysema, suggesting that inflammasome pathways are involved in the establishment of COPD (18, 19). Inflammasomes are cytoplasmic multiprotein complexes orchestrating diverse functions during homeostasis and inflammation (20–22). These complexes are generally composed of a Nod-like receptor (NLR), ASC adaptor molecule, and caspase-1 (Casp-1) or caspase-11 (Casp-11) proteases, where activation leads to pro-IL-1β and pro-IL-18 maturation and further to biologically active IL-1β and IL-18 pro-inflammatory cytokines (20–22).

NOD-like receptor family pyrin domain containing (NLRP)6 inflammasome composed of NLRP6, ASC, and Casp-1 has been shown to regulate microbiota homeostasis and antibacterial immunity in the intestine (23–25). NLRP6 inflammasome orchestrates goblet cell mucin granule exocytosis, as mucus accumulation and impaired secretion were observed in Nlrp6-deficient goblet cells (25). Moreover, recently identified intestinal sentinel goblet cells were shown to non-specifically recognize bacterial compounds leading to NLRP6 inflammasome activation and subsequent calcium-dependent mucin exocytosis from sentinel and adjacent goblet cells (26). In addition, NLRP6 expression in inflammatory monocytes reduces susceptibility to chemically induced intestinal injury (27). An inflammasome-independent NLRP6 role in the control of enteric virus infection was reported, where NLRP6 interacted with an RNA sensor, triggering type I and type III interferon-mediated antiviral responses (28). In contrast to the gut, the role of NLRP6 in lung inflammation and repair has been poorly evaluated. A few recent studies showed that this multifaceted innate immune sensor controls neutrophil recruitment and function during pulmonary gram-positive and gram-negative bacterial infections (29–31).

Here, we demonstrate for the first time that the NLRP6 inflammasome governs lung inflammation from CS by controlling neutrophilic inflammation. Impaired lung inflammation in CS-exposed Nlrp6-deficient mice is transferable to WT mice by co-housing experiments or fecal microbiota transplantation. Mechanistically, we show that gut microbiota from Nlrp6-deficient mice regulates lung CXCL5 production and neutrophil influx. Our data also indicate that CS exposure modulates gut microbiota composition in both WT and Nlrp6-deficient mice. In conclusion, we report that smoking induces an unexpected gut-to-lung axis modulating pulmonary inflammation, which depends on NLRP6 expression in lung and intestinal epithelial cells.

## Methods

### Mice

Wild-type C57BL/6J (WT) mice were purchased from Janvier Labs or bred in our animal facility for microbiota experiments together with gene deficient or conditional mouse strains. Asc^-/-^ mice were provided by Francis Derouet at Lausanne University (32), Casp-1/11^-/-^ by Seshadri Tara at BASF Bioresearch corporation (33), and Nlrp6^-/-^ by Mathias Chamaillard at Lille Pasteur Institute (24). Nlrp6 flox/flox mice were obtained from the Philip Rosenstiel Institute of Clinical Molecular Biology, Kiel, Germany. Nlrp6 tissue-specific knockouts were obtained by breeding Nlrp6 flox/flox with Aqp5Cre knock-in (Acid or Aqp5tm1.1(cre,DsRed)Pfl), allowing deletion in alveolar type I (AT1) cells in adult lung (34). All mouse strains were backcrossed 10 times or made on C57Bl/6J background and housed at the animal facility at the Transgenose Institute (UPS-TAAM) in CNRS Orleans, France. Except for co-housing experiments, 6–10-week-old mice were kept in sterile, isolated, and ventilated cages. All animal experiments followed the French government’s ethical and animal experiment regulations (CLE CCO 2015-1088).

### Cigarette smoke exposure

3R4F cigarettes (University of Kentucky) were used without filter. Mice were placed in an InExpose smoke chamber (EMKA Technologies) and inhaled smoke from four cigarettes, three times a day for 4 days for the acute inflammation model, or four cigarettes, three times a day, 5 days a week for 6 weeks for the subchronic inflammation model. Bronchoalveolar lavage (BAL) and lung tissue were collected about 16 hours after the last CS exposure.

### Broncho-alveolar lavage

BAL was performed as previously described (19). Differential cell counts were performed by counting an average of 250 cells on Cytospin preparations (Shandon CytoSpin 3, Thermo Fisher Scientific) after May-Grünwald-Giemsa staining (RAL-Diff Quick, Siemens) according to manufacturer’s instructions.

### Tissue homogenates

Lungs were perfused with Isoton (Beckman Coulter) to flush the vascular content. Washed lungs and ileum were homogenized by a rotor-stator (Ultra-turrax) in PBS with protease inhibitor cocktail (Roche) for mediator measurement or in RIPA buffer with protease inhibitor cocktail (Thermo Fisher Scientific) for immunoblotting analysis. Extracts were centrifuged and supernatants stored at –80°C.

### Lung histology

Lung left lobe was fixed in 4% buffered formaldehyde (MMFrance), processed, and paraffin embedded under standard conditions. Lung sections of 3 µm were stained with Direct Red 80 (Red Sirius, Sigma-Aldrich). The slides were blindly examined at 10-times magnification (Leica) and cell infiltration assessed by a semi-quantitative score (with increasing severity 0–5) by two independent investigators.

### Mediator measurements

BALF supernatants and lung homogenates mediators were measured by ELISA assay kits for murine MPO, IL-1β, CXCL1, CXCL5, CXCL15, BAFF, LCN2, MMP-9, and TIMP-1 (R&D System) according to manufacturer’s instructions.

### Air–liquid interface trachea epithelial cell culture

Mice tracheas were collected from wild type C57BL/6J (WT). Using pronase (1.5 mg/mL) (Roche®), tracheas were digested overnight at 4°C. Digestion was stopped using MTEC/Basic medium (Composition Table X) + 10% fetal bovine serum (FBS). The DNAase (0.5 mg/mL) (Sigma-Aldrich®) step was realized in MTEC/Basic medium without FBS. Adherent cells were removed by plating samples on specific petri dishes (Primaria Corning). Of the trachea epithelial cells, 33.10^3^ put in 150 µL of MTEC/Plus medium (Composition **Table 1**) were plated on the permeable membrane of Transwell inserts precoated with rat-tail collagen (0.05 mg/mL diluted in acetic acid 20 mM)(Thermo Fisher®) pre-coated Transwell. Then, MTEC/Plus medium was added in the basolateral chamber of Transwell inserts.

**Table 1:** Trachea epithelial cells culture medium compositions.

MTEC/Basic	Concentration
HEPES	15,000 mM
Pen/strep	1 X
MTEC/Plus	Concentration
Insulin Transferrin cocktail 1X (Thermo Fisher^®^)	10 µg/mL
Cholera toxin (Sigma-Aldrich^®^)	0.1 µg/mL
Epidermal Growth Factor (EGF) (Sigma-Aldrich^®^)	0.025 µg/mL
Bovine Pituitary Extract (Fisher Gibco^®^)	30 µg/mL
FBS	5%
Retinoic Acid B (RA-B) (Sigma-Aldrich^®^)	0.05 µM
MTEC/NS	Concentration
NuSerum	2%
Retinoic Acid B (RA-B) (Sigma-Aldrich^®^)	0.05 µM
MTEC/SF	Concentration
Insulin Transferrin cocktail 1X (Thermo Fisher^®^)	5 µg/mL
Cholera toxin (Sigma-Aldrich^®^)	0.025 µg/mL
Epidermal Growth Factor (EGF) (Sigma-Aldrich^®^)	5 ng/mL
Bovine Pituitary Extract (Fisher Gibco^®^)	30 µg/mL
Commercial BSA solution (sterile) (Sigma-Aldrich^®^)	1 mg/mL
Retinoic Acid B (RA-B) (Sigma-Aldrich^®^)	0.05 µM

At day 4, the apical medium was changed for MTEC/Plus medium until cell confluence. Basolateral medium was changed every day with MTEC/SF differentiation medium (Composition **Table 1**). At cell confluence, apical chamber medium was removed and cells were washed with phosphate buffered saline (PBS). Trachea epithelial cells in ALI were grown at 37°C in 5% of CO2 until cell collection or stimulation.

### Specific *Nlrp6* RT-PCR on trachea epithelial cells

Total RNA was extracted from epithelial cells using an isolation kit (RNeasy kit^®^) following the manufacturer’s protocol. RNA concentration and integrity were determined using a Nanodrop (Nd-1000) spectrophotometer (Labtech^®^). Reverse transcription of RNA into cDNA was carried out with GoScript™ Reverse Transcription System (Promega^®^). Using iScript RT supermix (BioRad^®^), 500 ng of total RNA was subjected to reverse transcription. PCR was performed on 8.75 µg of synthetized cDNA. Specific primers used for *Nlrp6* mRNA amplification were as follows: *Nlrp6* forward 5’-GAC-CAG-TTT-AGC-CCA-GAA-AAG-G-3’; *Nlrp6* reverse 5’-CTC-CAG-TGT-AGC-CAT-AAG-CAG- 3’. The PCR program is described in **Table 2**.

**Table 2:** Steps within the PCR program.

Initialization	94°C, 2 min			
	5 cycles	5 cycles	5 cycles	5 cycles
Denaturation	94°C, 30 s	94°C, 30 s	94°C, 30 s	94°C, 30 s
Annealing	62°C, 30 s	61°C, 30 s	59°C, 30 s	58°C, 30 s
Elongation	72°C, 1 min	72°C, 1 min	72°C, 1 min	72°C, 1 min

### FLAG immunohistochemistry

Lungs were fixed with 4% paraformaldehyde (Sigma-Aldrich®) for 72 h, embedded in paraffin, and divided into sections of 3 µm. Lung sections were dewaxed and rehydrated, then heated 20 min at 80°C in citrate buffer of 10 mM pH=6 for antigen retrieval (unmasking step). Lung sections were permeabilized in PBS 0.5% triton X-100, blocked with 5% FCS for 1 h at room temperature, and then incubated overnight with primary mouse anti-FLAG (1:100, A9542 Sigma). After washing, sections were incubated with the appropriate second antibody conjugated with horseradish peroxidase (1:200 anti-mouse IgG, Sigma-Aldrich^®^) in 1% FCS for 1 h at room temperature. Following washing, lung sections were incubated with HRP Substrate, DAB (Vector Laboratories^®^), following the manufacturer’s protocol. After distilled water washing, Gill hematoxylin counterstaining on lung sections was done. Then, lung sections were dehydrated, and fixed and mounted onto microscope slides (Eukitt). Slides were examined using a scanner in NDP view.

### Treatments

Mice were anesthetized by an intra-muscular injection of ketamine (10 mg/mL, Merial) and xylazine (0.2%, Bayer). Recombinant murine CXCL5/LIX (9-78 amino acid, 1 µg per mouse) was administered intranasally at days 2 and 4 between the second and the third daily CS exposure. Anti-CXCL5/LIX antibody was obtained from Jacques Van Snick. 150 µg per mouse was injected intraperitoneally at days 2 and 4 between the second and the third daily CS exposure. An antibiotic cocktail containing Vancomycin 0.5 g/L (Sandoz), Ampicillin 1 g/L (Euromedex), Neomycin 1 g/L (Euromedex), Metronidazole 1 g/L, and sucrose 1% (Sigma-Aldrich) diluted in sterile water was given in drinking water and replaced every 3 days.

### Generation of bone marrow chimeras

CD45.1 WT and CD45.2 Nlrp6^-/-^ recipient mice were lethally irradiated (35), and 4x10^6^ NLRP6-deficient or WT bone marrow cells were injected into the lateral tail vein 24 h afterwards. Four chimeric mouse groups were obtained: WT>WT, NLRP6^-/-^>WT, WT>NLRP6^-/-^, to put before and NLRP6^-/-^ >NLRP6^-/-^. Bone marrow reconstitution was controlled by flow cytometry assessing the ratio of CD45.1 versus CD45.2 blood immune cells. Three months after bone marrow reconstitution, mice were exposed to air or CS as described.

### Co-housing experiments

Immediately after weaning, WT and Nlrp6^-/-^ gender-matched animals were co-housed at 1:1 ratio for 12 weeks, and mice were exposed to air or CS as described.

### Fecal transplantation

Fecal transplantation was performed as previously described (35). Briefly, 3-week-old germ-free C57BL6/J (WT) mice generated in-house in sterile isolators (TAAM-CNRS) were orally transplanted with 200 µl fecal homogenates from SPF-WT or SPF-Nlrp6^-/-^ mice. Mice were maintained in an isolator until 7 weeks of age, placed in a conventional breeding facility for one week for adaptation, and exposed to CS for 4 days.

### Quantitative RT-PCR

Total RNA was isolated from homogenized mouse lung using Tri Reagent (Sigma-Aldrich) and quantified by NanoDrop (Nd-1000). Reverse transcription of RNA into cDNA was carried out with GoScript™ Reverse Transcription System (Promega). RT-qPCR was performed with Fast GoTaq qPCR Master Mix (Promega) on ARIA MX (Agilent Technologies). *Mmp12* primers were purchased from Qiagen. RNA expression was normalized to *Gapdh* (Qiagen) expression and analyzed using the ^ΔΔ^Ct method.

### Immunoblotting

Protein concentrations in tissue homogenates were determined using Pierce BCA protein assay (Thermo Fisher Scientific). Proteins amounting to 40 µg were denatured by boiling (95°C, 5 min) in reducing SDS sample buffer, separated by SDS-PAGE, and transferred to nitrocellulose membranes (GE Healthcare). Membranes were blocked 2 hours in 5% Blotting-Grade Blocker (BioRad), washed three times in Tris-buffered saline (TBS)- 0.1% Tween 20, and incubated with primary rabbit anti-murine LIX antibody (Peprotech) overnight at 4°C. Membranes were washed three times in TBS- 0.1% Tween 20 and incubated with the appropriate secondary antibody conjugated with horseradish peroxidase (HRP) for 1 hour at room temperature. Membranes were incubated with mouse anti-actin HRP-conjugate (Sigma-Aldrich) in 5% Blotting-Grade Blocker in TBS-0.1% Tween 20 for 1 hour at room temperature. Detection was performed with ECL Prime Western-blotting Detection Reagent (GE Healthcare) and multi-application gel imaging system PXi software (Syngene).

### Immunostaining

Lungs and ileum were fixed in 4% paraformaldehyde (PFA) (Sigma-Aldrich) and then dehydrated in 30% sucrose (Sigma-Aldrich) solution for 2 weeks. Organs were embedded in Tissue-Tek® OCT™ (Sakura) and stored at –80°C prior to being sliced. Sections were incubated for 30 minutes in pre-heated antigen retrieval buffer (Citrate buffer 10 mM pH=6). Sections were incubated in TBS-Triton X-100 0.1% and then in blocking solution containing 1% bovine serum albumin (BSA) -10% fetal bovine serum (FBS) -0.1% Triton X-100 in TBS. Primary antibody directed against CXCL5 (Peprotech) was incubated in blocking solution overnight at 4°C. Sections were rinsed three times in TBS and incubated with appropriated secondary antibody. Slides were counterstained using 4′,6-diamidino-2-phenylindole (DAPI) for 10 minutes, rinsed, and coverslips were mounted with Fluoromount-G medium (SouthernBiotech). Images were treated using ImageJ software.

### Stool collection and DNA extraction

Feces were collected and immediately frozen at –80°C for further analysis. DNA was extracted from the fecal samples as described (36). Following microbial lysis by both mechanical and chemical methods, nucleic acids were precipitated in isopropanol for 10 minutes at room temperature, incubated for 15 minutes on ice, and centrifuged for 30 minutes at 20,000 g at 4°C. Pellets were resuspended in 450 μL of phosphate buffer and 50 μL of potassium acetate. After RNase treatment and DNA precipitation, nucleic acids were recovered via centrifugation at 20,000 g at 4°C for 30 minutes. The DNA pellet was resuspended in trypsin-EDTA buffer. DNA samples were then subjected to 16S sequencing.

### 16S rRNA sequencing

Bacterial diversity in stools was determined by targeting a portion of the ribosomal genes in extracted DNA. A 16S rRNA gene fragment comprising the V3 and V4 hypervariable regions (16S sense 5′-TACGGRAGGCAGCAG-3′ and antisense 5′-CTACCNGGGTATCTAAT-3′) was amplified using an optimized and standardized 16S-amplicon-library preparation protocol (Metabiote, GenoScreen, Lille, France). Briefly, 16S DNA PCR was performed using 5 ng of genomic DNA according to the manufacturer’s protocol (Metabiote), 192 bar-coded primers (Metabiote MiSeq Primers) at final concentrations of 0.2 μmol/L, and an annealing temperature of 50°C for 30 cycles. The PCR products were purified using an Agencourt AMPure XP-PCR purification system (Beckman Coulter, Brea, CA, USA), quantified according to the manufacturer’s protocol, and multiplexed at equal concentrations. Sequencing was performed using a 300-bp paired-end sequencing protocol on an Illumina MiSeq platform (Illumina, San Diego, CA, USA) at GenoScreen, Lille, France. Raw paired-end reads were subjected to the following processes: (1) quality filtering using the PRINSEQ-lite PERL script (Schmieder R, Edwards R. Quality control and preprocessing of metagenomic datasets. Bioinformatics 2011;27:863-4), by truncating the bases from the 3′ end that did not exhibit a quality <30, based on the Phred algorithm; (2) paired-end read assembly using fast length adjustment of short reads to improve genome assemblies (FLASH) (Magoc T, Salzberg SL. FLASH: fast length adjustment of short reads to improve genome assemblies. Bioinformatics 2011;27:2957-63.) with a minimum overlap of 30 bases and a 97% overlap identity; and (3) searching for and removing both forward and reverse primer sequences using CutAdapt, with no mismatches allowed in the primer sequences. Assembled sequences, for which perfect forward and reverse primers were not found, were eliminated.

### 16S rRNA sequence analysis

The sequences were demultiplexed and quality filtered using the QIIME version 1.9.1 software package (37). The sequences were then assigned to operational taxonomic units (OTUs) using the UCLUST algorithm with a 97% pairwise identity threshold and classified taxonomically using the Greengenes reference database (version 13.5). Rarefaction was performed (32,000 sequences per sample) and used to compare the relative abundance of OTUs across samples. Beta diversity was measured by a Bray Curtis distance matrix and was used to build principal coordinate analysis (PCoA) plots. Raw sequence data are accessible in the European Nucleotide Archive.

### Statistical analysis

Statistical evaluation of differences between experimental groups was determined by one-way ANOVA, analysis of variance, and Bonferroni test for *in vivo* experiments using Prism software (La Jolla, CA, USA). *P* values <0.05 were considered statistically significant. All tests were performed with Graphpad Prism, Version 8 for Windows (GraphPad Software Inc.). Data are expressed as mean ± SEM. Statistically significant differences were defined as follows: *P<0.05, **P<0.01, ***P<0.001 and****P<0.0001.

## Results

### Nlrp6 deficiency dampens lung inflammation and remodeling following CS exposure

**Figure 1:**
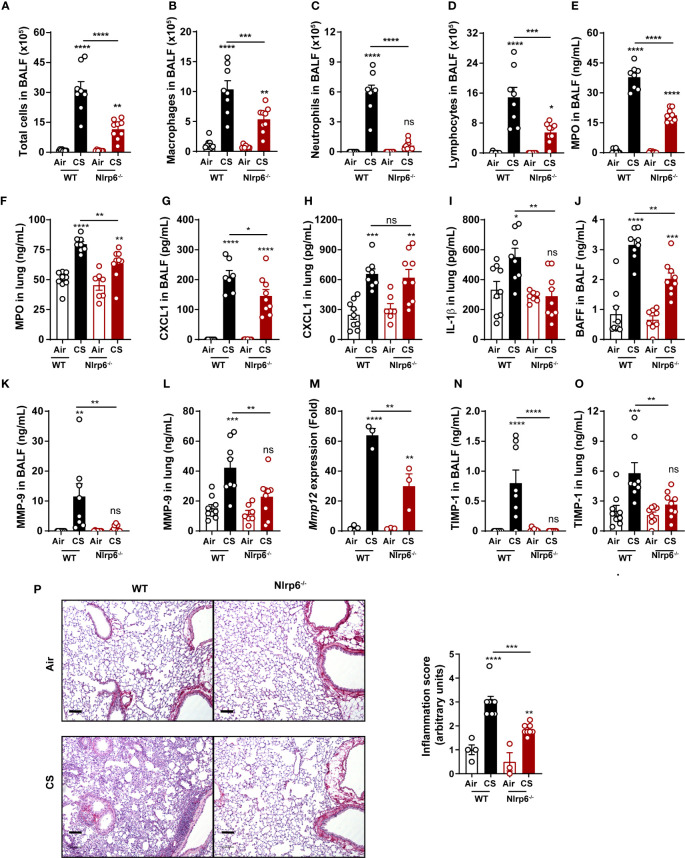
NLRP6 deficiency impairs inflammation and remodeling factors to subchronic cigarette smoke exposure. C57BL/6 (WT) mice and Nlrp6^-/-^ mice were exposed to air or cigarette smoke (CS) for 6 weeks. BALF and lungs were collected 16 hours after the last exposure. **(A)** Total cells, **(B)** macro- phages, **(C)** neutrophils and **(D)** lymphocytes were numerated in BALF. **(E, F)** Myeloperoxydase (MPO) and **(G, H)** CXCL1 levels were measured in BALF and lungs. **(I)** IL-1β and **(J)** BAFF protein levels were analyzed in lungs and BALF respectively. **(K, L)** MMP-9 levels in BALF and lungs were measured by ELISA. **(M)** Mmp12 mRNA expression was analyzed by qPCR in lung homogenates. **(N, O)** TIMP-1 levels in BALF and lungs were measured. **(P)** Lung sections were stained with Red Sirius and inflammation score was graphed ). Scale bar = 100μm. Data are representative of one experiment and are expressed as mean values ± SEM and n= 6-9 mice per group, ns, non-significant, *p < 0.05, **p < 0.01, ***p < 0.001, ****P<0.0001.

Since NLRP6 was shown to regulate intestinal epithelium homeostasis and immunity (24, 25, 28), we wanted to investigate whether it could control pulmonary epithelium function and airway inflammation following cigarette smoke (CS) exposure. WT and Nlrp6-deficient (Nlrp6^-/-^) mice were subchronically exposed to CS three times a day, 5 days a week, for 6 weeks. Compared to unexposed (air) mice, subchronically-exposed WT mice (CS) displayed elevated numbers of bronchoalveolar fluid (BALF) cells (**Figure 1A**), mainly macrophages (**Figure 1B**), neutrophils (**Figure 1C**), and lymphocytes (**Figure 1D**), which were all strongly attenuated in CS-exposed Nlrp6^-/-^ mice. Among immune cells, neutrophils play a major role in response to CS (38, 39). As a marker of neutrophil recruitment, myeloperoxidase (MPO) was significantly reduced in the BALF and the lungs (**Figures 1E, F**) of CS-exposed Nlrp6^-/-^ mice. In comparison, neutrophilic chemokine CXCL1 production was significantly decreased in the BALF of Nlrp6^-/-^ mice (**Figure 1G**) but not in lung homogenates (**Figure 1H**). Since NLRP6 was shown to regulate IL-1β and IL-18 production through inflammasome activation, we analyzed IL-1β lung expression and showed that its CS-mediated induction was significantly attenuated in Nlrp6^-/-^ mice (**Figure 1I**). In addition, as a correlate of B lymphocyte recruitment and disease severity (40), B cell activating factor (BAFF) induction following CS-exposure was significantly reduced in the BALF of Nlrp6^-/-^ compared to WT mice (**Figure 1J**).

We next measured proteases involved in COPD establishment and in particular matrix metalloproteinase (MMP) and their inhibitors (TIMP). MMP-9 protein levels in BALF and lungs (**Figures 1K, L**), *Mmp-12* mRNA expression in lungs (**Figure 1M**), and tissue inhibitor of MMP (TIMP)-1 protein levels in BALF and lungs (**Figures 1N, O**) were significantly decreased in subchronically CS-exposed Nlrp6^-/-^ mice compared to WT mice. Lung microsections and histology analysis showed reduced inflammation in Nlrp6^-/-^ mice in comparison to WT mice (**Figure 1P**). In addition, acute CS exposure over 4 days confirmed reduced inflammation in Nlrp6^-/-^ in comparison to WT mice. While total BAL cells numbers were not significantly changed (**Figure S1A**), we report a strong reduction of neutrophils (**Figure S1B**), MPO levels (**Figure S1C**) in the BALF, and IL-1β levels (**Figure S1D**) but not IL-18 (**Figure S1E**) in the lungs. BALF levels of CXCL1/KC were significantly reduced in Nlrp6^-/-^ mice (**Figure S1F**) but not in lung homogenates (**Figure S1G**) as observed upon subchronical exposure. We also observed reduced MMP-9 levels in BALF and lungs (**Figures S1H, I**), *Mmp-12* mRNA expression in lungs (**Figure S1J**), and TIMP-1 protein levels in BALF and lungs (**Figures S1K, L**) in Nlrp6^-/-^ mice compared to WT mice. Lung microsections and histology analysis showed reduced inflammation in Nlrp6^-/-^ mice in comparison to WT mice (**Figure S1M**).

In addition, to verify that mouse genetic background and/or housing were not responsible for the difference in inflammatory response to CS observed, we performed breeding between Nlrp6^+/-^ mice in order to obtain Nlrp6^+/+^ and Nlrp6^-/-^ littermates (**Figure S2A**), and we acutely exposed them to CS. Importantly we confirmed that neutrophil number and percentage in BALF (**Figures S2B, C**), MPO levels in lungs and BALF (**Figures S2D–E**), IL-1β in lungs (**Figure S2F**), CXCL1/KC, and CXCL5/LIX in BALF and lungs were (**Figures S2G–J**) in Nlrp6^-/-^ mice compared to WT mice.

Altogether, our results demonstrate that NLRP6 is central to pulmonary inflammatory responses to CS exposure. Since NLRP6 plays comparable roles in pulmonary inflammation and remodeling upon acute (1 w) and subchronic (6 w)-CS exposure in mice, we then preferentially performed acute exposure to investigate the mechanism of NLRP6-mediated lung inflammation.

### NLRP6 expression in radioresistant cells is necessary for neutrophil influx and IL-1β production

**Figure 2:**
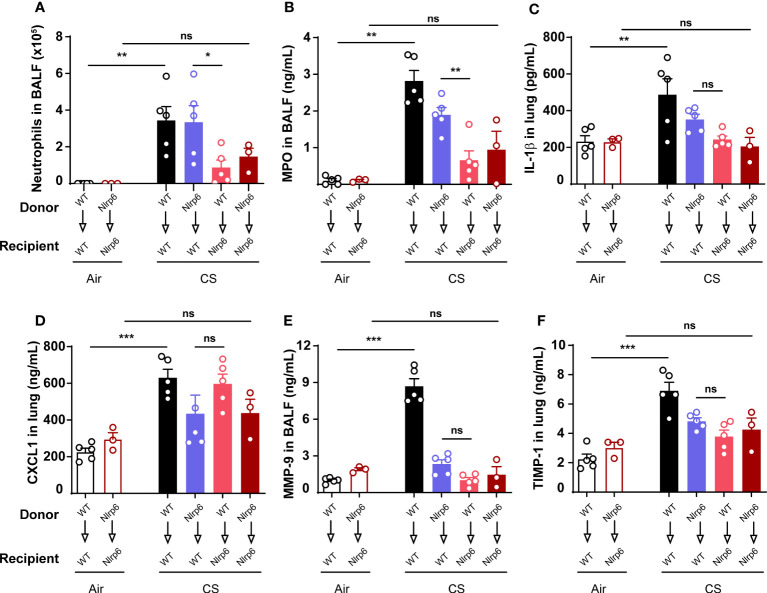
Nlrp6 expression in non-immune/resident cells is necessary for neutrophils influx upon CS exposure. WT (CD45.1) and Nlrp6^-/-^ (CD45.2) recipient mice were sub-lethally irradiated, reconstituted by either WT or Nlrp6^-/-^ BM cells and exposed to air or CS for 4 days. BALF and lungs were collected 16 hours after the last exposure. **(A)** Neutrophils were numerated in BALF. **(B-D)** MPO in BALF, IL-1β and CXCL1 levels in lungs, **(E)** remodeling factors MMP-9 in BALF and **(F)** TIMP-1 in lungs were assessed by ELISA. Data are representative of two experiments and are expressed as mean values ± SEM (n= 3-5 mice per group), ns, non-significant, *p < 0.05, **p < 0.01, ***p < 0.001.

In order to investigate the cellular source of NLRP6 involved in acute CS-induced inflammation, we addressed its respective contribution in bone marrow (BM)-derived versus resident cells using bone marrow transplantation. WT (CD45.1) and Nlrp6^-/-^ (CD45.2) recipient mice were sub-lethally irradiated (2 x 5.5 Gy, 3 h apart) and reconstituted with either WT or Nlrp6^-/-^ BM cells. CS-induced neutrophil recruitment (**Figure 2A**) and MPO levels (**Figure 2B**) in BALF were significantly reduced in Nlrp6-deficient recipient mice (WT>Nlrp6^-/-^ and Nlrp6^-/-^>Nlrp6^-/-^) but not in WT recipient mice (Nlrp6^-/-^>WT and WT>WT). In addition, we observed a trend for decreased lung IL-1β levels in Nlrp6-deficient recipient mice (**Figure 2C**). These data indicate that NLRP6-dependent neutrophil influx, MPO, and potentially IL-1β production in response to acute CS-exposure are dependent on Nlrp6 expression by non-immune/radioresistant cells rather than BM-derived immune cells. In addition, while not significantly decreased in Nlrp6^-/-^>Nlrp6^-/-^ and Nlrp6^-/-^>WT mice, lung CXCL1 expression seems to account for a non-negligible BM-derived contribution (**Figure 2D**). In addition, remodeling factors MMP-9 (**Figure 2E**) and TIMP-1 (**Figure 2F**) were decreased in Nlrp6^-/-^>WT, WT>Nlrp6^-/-^, and Nlrp6^-/-^>Nlrp6^-/-^ mice in comparison to WT>WT mice, suggesting an NLRP6-dependent production in both BM-derived and resident cells. As a whole, these data indicate that BALF neutrophilic influx upon CS exposure depends on NLRP6 expression in resident cells.

### Acute CS exposure induces NLRP6 expression in airway epithelial cells

**Figure 3:**
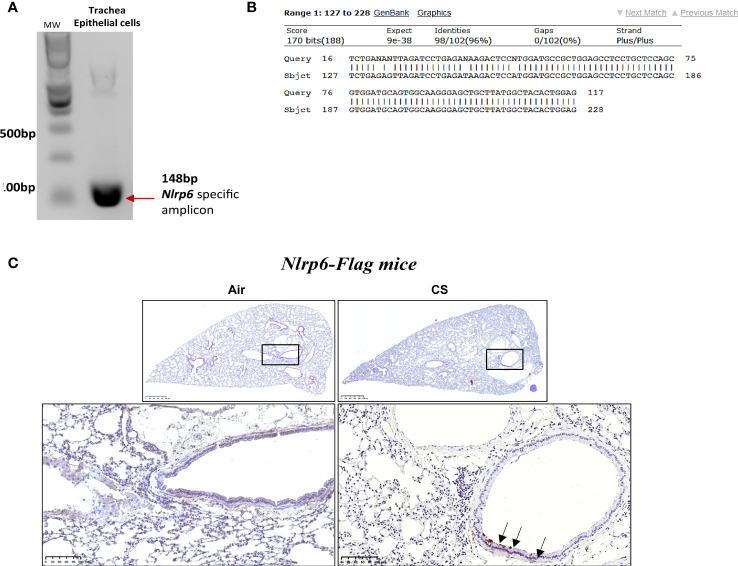
*Nlrp6* mRNA basal expression in trachea epithelial cells *(in vitro)* and NLRP6 induced protein expression in bronchial cells *(in vivo)* upon cigarette smoke exposure. Agarose DNA gel showing RT-PCR product of Nlrp6 sequence **(A)**. The representative sequencing result of trachea epithelial cells detected band showing the correct Nlrp6 cDNA **(B)**. Epithelial cells markers (*Krt8* and *Oclnl*) were quantified by qPCR **(C)**. FLAG immunohistochemistry on Nlrp6-FLAG-IRES-GFP mice exposed to cigarette smoke (CS) or air during 4 days CS exposure led to Nlrp6 expression in bronchial cells **(D)**. Data are representative of 2 experiments and are expressed as mean values ± SEM.

Since we observed that neutrophil influx upon CS-exposure depends on NLRP6 expression in resident cells (**Figures 2A, B**), we investigated whether NLRP6 is expressed in epithelial cells. We first isolated trachea epithelial cells from wild-type mice and performed air–liquid interface (ALI) in *in vitro* culture. We analyzed Nlrp6 mRNA basal expression by specific *Nlrp6* RT-PCR and observed a 148 bp band corresponding to expected Nlrp6 cDNA size (**Figure 3A**). Epithelial cells markers (*Krt8* and *Ocln1*) were quantified by qPCR, confirming basal Nlrp6 expression in trachea epithelial cells (not shown). Sequencing this amplified cDNA, we identified Nlrp6 sequence (**Figure 3B**). Then we exposed NLRP6-FLAG-IRES-GFP reported mice and WT C56BL/6 mice to CS or air for 4 days. Performing FLAG-specific immunohistochemistry analysis of lung sections, we observed increased FLAG expression in bronchial cells from CS-exposed mice in comparison to air-exposed mice and no FLAG expression in WT C56BL/6 CS-exposed mice (**Figure 3C**). These results indicate that acute CS-exposure enhances NLRP6 expression in bronchial airway epithelial cells in mice.

### Specific NLRP6 deficiency in lung cells dampens pulmonary inflammation following CS exposure

**Figure 4:**
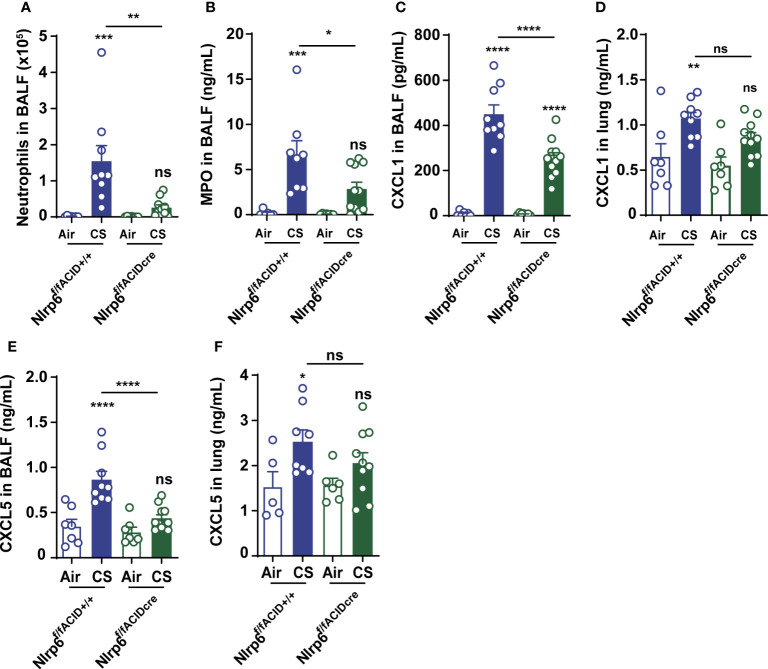
NLRP6 expression in airway epithelial cells controls inflammation and CXCL5 secretion to CS. Mice deficient for Nlrp6 specifically in AEC (Nlrp6^f/f^ Acid^+/CRE^) as well as their controls (Nlrp6ff^f/f^ Acid^+/+^) were exposed to air or CS for 4 days. BALF and lungs were collected 16 hours after the last exposure. **(A)** BALF Neutrophil counts. **(B)** BALF MPO levels. **(C-F)** CXCL1 and CXCL5 levels measured in BALF and lungs. Data are representative of three experiments and are expressed as mean values ± SEM (n= 7-9 mice per group), ns, non-significant, *p < 0.05, **p < 0.01, ***p < 0.001. ****P<0.0001.

To confirm that NLRP6 expressed in lung cells may control airway and lung inflammation, we generated mice deficient for Nlrp6, specifically in aquaporin-expressing lung epithelium (Nlrp6^fl/fl^ Acid^+/CRE^), as well as their control littermate mice (Nlrp6^fl/fl^ Acid^+/+^), and exposed them to CS for 4 days. We showed that Nlrp6^fl/fl^ Acid^+/Cre^ mice displayed reduced BALF total cells influx and, in particular, neutrophil counts and MPO levels in comparison to Nlrp6^fl/fl^ Acid^+/+^ littermate mice (**Figures 4A, B**). CXCL1 (**Figures 4C, D**) and CXCL5 (**Figures 4E, F**) levels were very significantly decreased in the BALF, whereas only a tendency was observed in the lungs of CS-exposed Nlrp6^fl/fl^ Acid^+/Cre^ as shown above in total Nlrp6^-/-^ mice. Altogether, our findings indicate that NLRP6 expression in lung cells controls pulmonary inflammation from CS exposure.

### NLRP6 inflammasome controls pulmonary inflammation and remodeling following CS exposure

**Figure 5:**
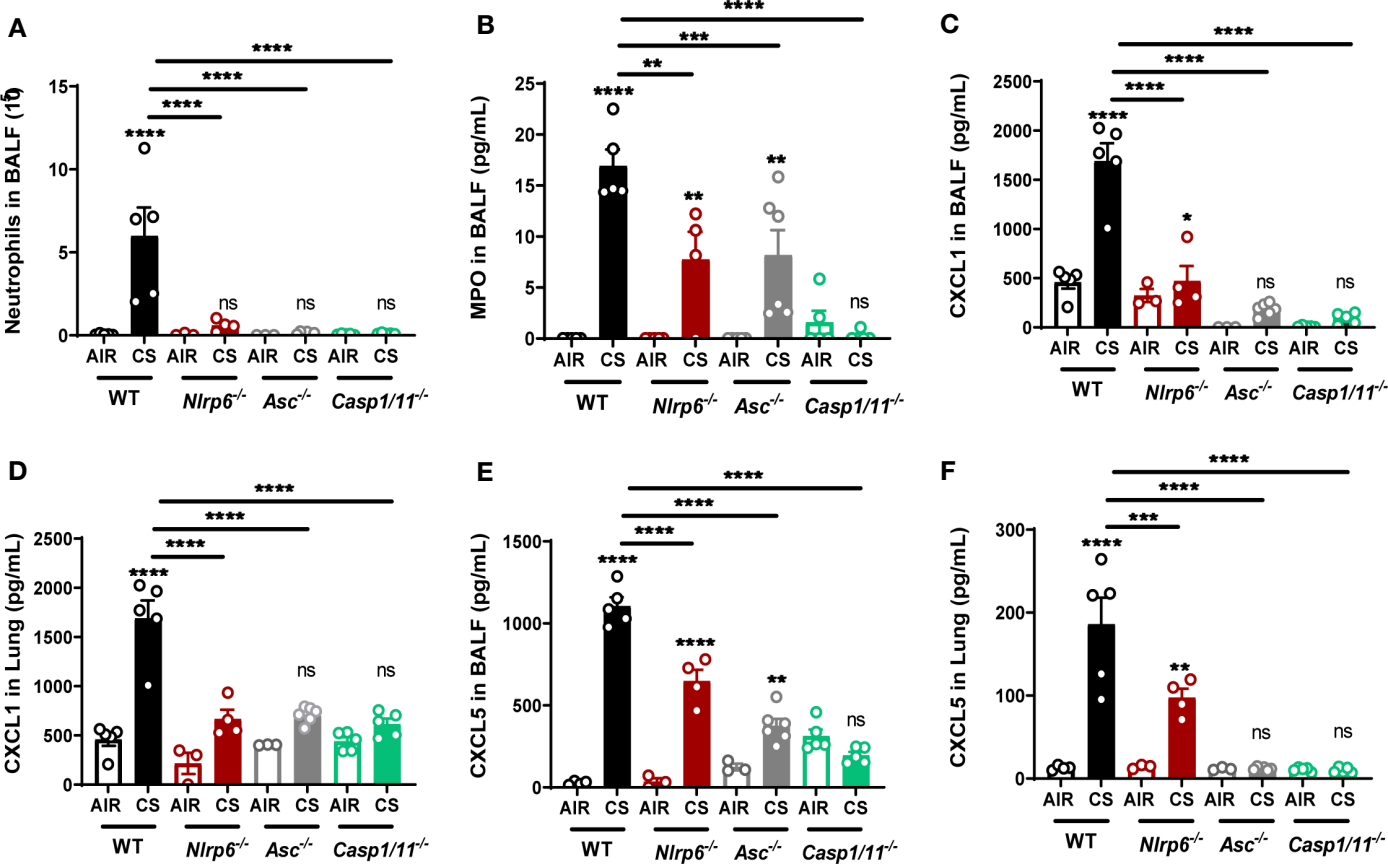
Nlrp6 deficiency leads to tenuated lung inflammation acumulation of CXCL5 in bronchial. WT, ASC^-/-^, Asc^-/-^, Casp1/11^-/-^ and Nlrp6^-/-^ mice were exposed to air or CS for 4 days. BALF and lungs were collected 16 hours after the last exposure. **(A)** Neutrophil numbers and **(B)** MPO levels were analyzed in BALF. **(C, D)** CXCL1 and **(E, F)** CXCL5 levels were measured in BALF and lungs, respectively. Data are representative of three experiments and are expressed as mean values + SEM (n= 4-5 mice per group), ns, non-significant, *p < 0.05, **p < 0.01, ***p < 0.001, ****P<0.0001.

Since part of NLRP6 function relies on inflammasome activation, we investigated the role of inflammasome-related molecules ASC, Casp-1, and Casp-11. After 4 days of CS exposure, we confirmed that BALF neutrophil numbers (**Figure 5A**), BALF MPO levels (**Figure 5B**), and CXCL1 and CXCL5 levels in BALF and lungs (**Figures 5C–F**) were dramatically reduced in Asc^-/-^ or Casp-1/11^-/-^ similar to what was observed in Nlrp6^-/-^ mice, suggesting an inflammasome-dependent function of NLRP6 in neutrophilic inflammation.

Since we observed that resident pulmonary cells play an important role in NLRP6 inflammasome-mediated airway inflammation following CS (**Figure 2**), we analyzed in more detail the role of CXCL5, a neutrophilic chemokine essentially produced by airway epithelial cells. We report that CXCL5 levels, as well as CXCL1, were decreased in both BALF (**Figures 5C, E**) and lungs (**Figures 5D, F**) of Asc^-/-^ or Casp-1/11^-/-^.

To confirm that CXCL5, essentially produced by airway epithelial cells, is a crucial chemokine in CS-induced pulmonary inflammation (11, 12, 38, 39), WT mice were intraperitoneally treated with CXCL5 neutralizing antibodies. A very significant decrease in BALF CXCL5 levels after anti-CXCL5 antibody treatment indicated efficient neutralization in the BALF (**Figure S3A**). Of note, we also observed decreased BALF CXCL1 levels, suggesting that CXCL5 secretion indirectly influences CXCL1 in CS-exposed mice (**Figure S3B**), supposedly by decreasing neutrophil-mediated macrophage activity. In line with decreased neutrophilic chemokines, BALF total cells, neutrophil numbers (**Figures S3C, D**), and MPO levels (**Figure S3E**) were reduced after CXCL5 blockade. Conversely, airway instillation of recombinant CXCL5 (rCXCL5) in CS-exposed Nlrp6^-/-^ mice was sufficient to restore most of CS-induced BALF neutrophil influx and MPO levels (**Figures S3F, G**), demonstrating that CXCL5 is a major neutrophil chemokine in CS-induced inflammation.

### Oral antibiotic treatment decreases pulmonary inflammation following CS exposure

**Figure 6:**
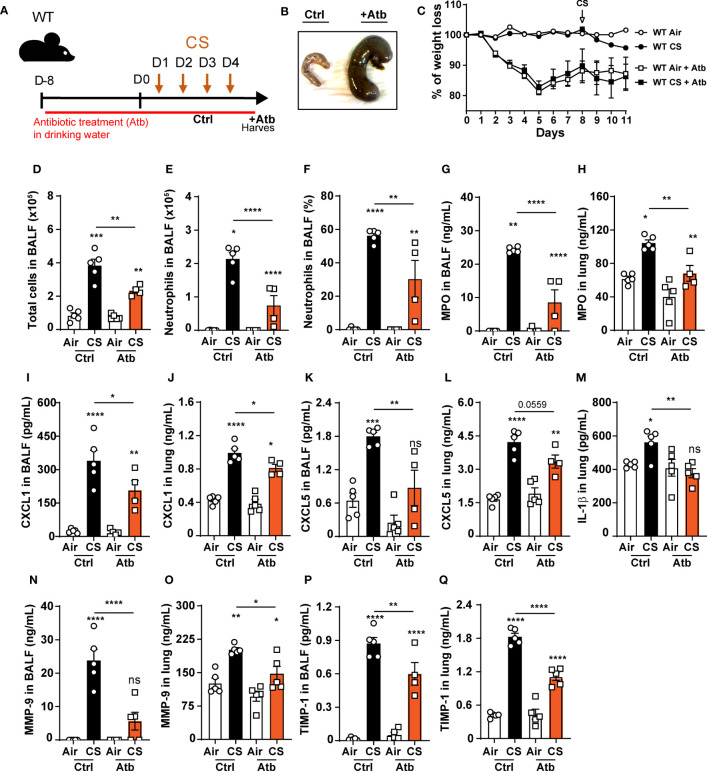
Oral antibiotic treatment of WT mice decreases pulmonary inflammation to cigarette smoke exposure. **(A)** WT mice were treated with antibiotic cocktail containing vancomycin, neomycin, ampicillin and me- tronidazole in drinking water for 12 days including the 4 days of CS exposure. BALF, lung and caecum were collected 16 hours after the last exposure. **(B)** Representative caecum picture from control (Ctrl) or treated (+Atb) mice. **(C)** Weight loss percentage **(D)**. Total cells and **(E)** neutrophils numerated in BALF. **(F)** BALF neutrophil percentage. **(G-L)** MPO, CXCL1, CXCL5 measured in BALF and lungs. **(M)** IL-1β levels in lungs. **(N-Q)** Remodeling factors MMP-9 and TIMP-1 levels measured in BALF and lungs. Data are representative of two experiments and are expressed as mean values ± SEM (n= 5 mice per group), *p < 0.05, **p < 0.01, ***p < 0.001, ****P<0.0001.

Since NLRP6 was shown to control gut microbiota composition and inflammation with dysbiosis in Nlrp6^-/-^ mice (25–27, 41, 42), we hypothesized that gut microbiota from Nlrp6^-/-^ mice could influence pulmonary inflammation through a gut-to-lung axis. In that context, we analyzed whether microbiota perturbation may disturb CS-induced lung inflammatory response and in particular neutrophilic chemokine secretion and neutrophil influx. We treated WT mice with broad-spectrum antibiotics in drinking water ad libitum for two weeks and exposed them to CS during the last 4 days (**Figure 6A**). Antibiotic treatment resulted in a drastic increase of caecum size (**Figure 6B**), as previously observed in germ-free mice, as well as significant body weight loss (**Figure 6C**). Total cell counts (**Figure 6D**), neutrophils percentage and numbers (**Figures 6E, F**) in BALF, MPO (**Figures 6G, H**), CXCL1 (**Figures 6I, J**), and CXCL5 (**Figures 6K, L**) levels were decreased in BALF and lung homogenates. In addition, IL-1β levels in the lungs (**Figures 6M**), and MMP-9 (**Figures 6N, O**) and TIMP-1 (**Figures 6P, Q**) levels in BALF and lungs were attenuated. These results show that whole body bacterial depletion in WT mice impairs pulmonary inflammation upon CS exposure and recapitulates our results with regard to Nlrp6^-/-^ mice. The data suggest that gut microbiota are involved in CS-induced lung inflammation.

### Impaired CS-induced airway inflammation in Nlrp6^-/-^ mice is transferable to wild-type mice by co-housing

**Figure 7:**
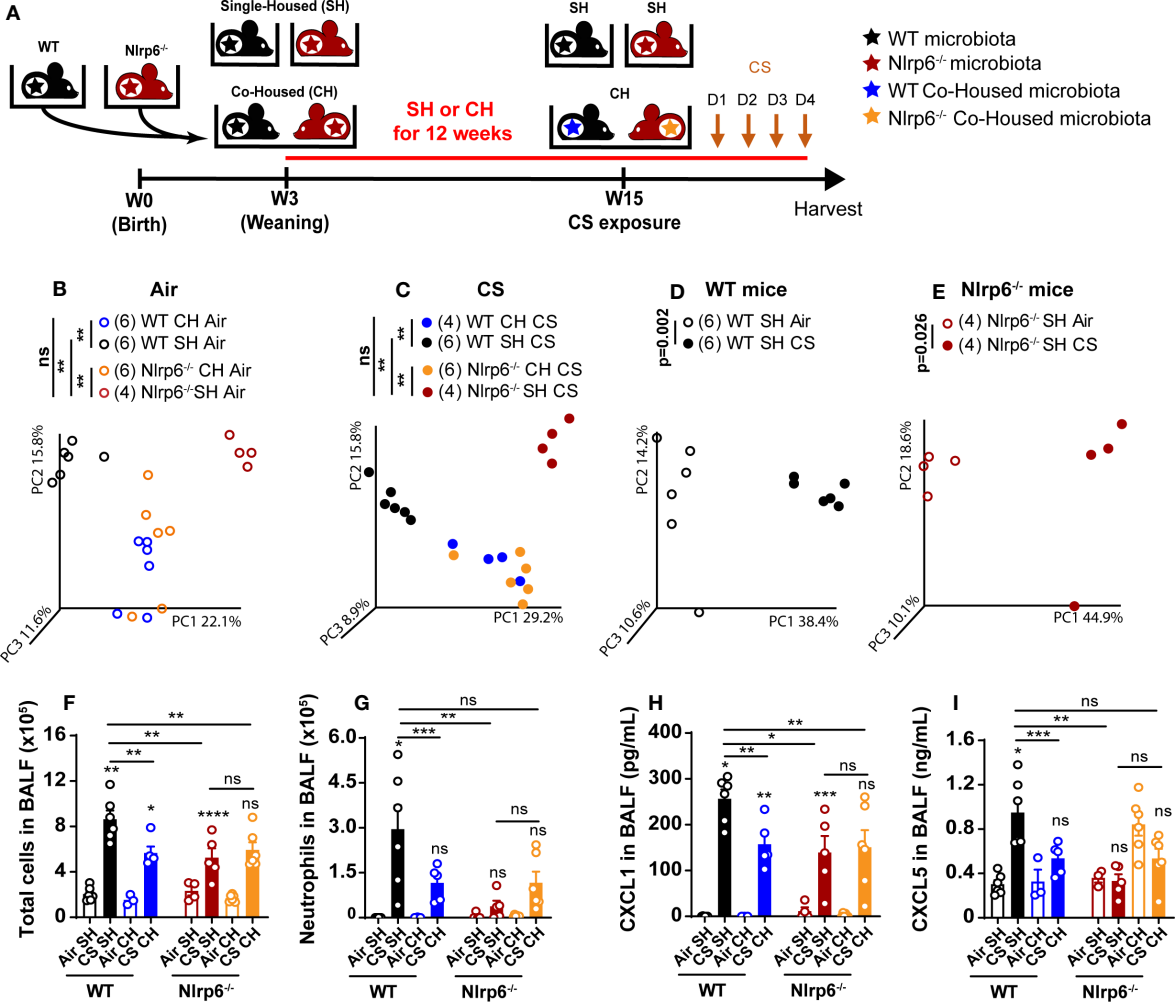
Transfer of impaired cigarette smoke-induced airway inflammation of Nlrp6^-/-^ mice to wild type mice by cohousing. **(A)** WT mice and Nlrp6^-/-^ mice were single-housed (SH) or co-housed (CH) for 12 weeks and exposed for 4 days of CS. BALF, lung and caecum were collected 16 hours after the last exposure. Principal components analysis (PCA) analysis of WT and Nlrp6^-/-^ SH or CH in **(B)** air or **(C)** CS conditions, **(D)** WT SH in air and CS conditions, and **(E)** Nlrp6^-/-^ SH in air and CS conditions. **(F)** BALF total cells and **(G)** neutrophils. **(H)** BALF CXCL5 and **(I)** CXCL1 levels. Data are representative of tywo expirements and are expressed as mean values ± SEM and n= 6-9 mice per group, ns, non significant, group), *p < 0.05, **p < 0.01, ***p < 0.001, ****P < 0.0001.

To decipher whether gut microbiota from Nlrp6^-/-^ influences pulmonary inflammation, WT mice were co-housed (WT CH) for 3 months with Nlrp6^-/-^ mice (Nlrp6^-/-^ CH). Single-housed WT (WT SH) and Nlrp6^-/-^ (Nlrp6^-/-^ SH) mice were used as controls. Then, mice were exposed to air or CS for 4 days (**Figure 7A**), and their gut microbiota composition was analyzed using unbiased 16S ribosomal RNA (rRNA) gene high throughput sequencing. As expected, the dominant phyla were represented by Firmicutes, Bacteroidetes, Proteobacteria, and Actinobacteria, and differences appeared between WT and Nlrp6^-/-^ mice (**Figures S4**, **S5**). No significant change in alpha diversity was observed in intestinal microbiota among the different mouse groups (**Figure S6**). However, principal component analysis (PCA) of beta diversity showed sharp differences comparing WT SH and Nlrp6^-/-^ SH mice, which were abrogated upon co-housing. This confirms differences in gut microbiota composition between WT and Nlrp6^-/-^ mice as previously reported (25, 41, 43, 44). Indeed, WT SH and Nlrp6^-/-^ SH mice exposed to air (**Figure 7B**) or to CS (**Figure 7C**) clustered separately on the PC1 axis, while the co-housed clusters became indistinguishable. Using LDA Effect Size (LEfSe) (45), specific differences stood out in gut microbiota composition between WT and Nlrp6^-/-^ mice exposed to air or CS (**Figures S4**, **S5**, **S7**). These differences mostly disappeared upon co-housing (**Figure S8**), demonstrating the efficacy of the microbiota transfer by this method.

In addition, analysis of gut microbiota from single-housed WT or Nlrp6^-/-^ mice exposed to air or to CS revealed that CS exposure affects intestinal microbiota composition in both WT (**Figure 7D**) and Nlrp6^-/-^ (**Figure 7E**) mice, indicating that CS airway exposure may influence gut homeostasis and/or inflammation. However, CS-driven gut microbiota alterations were distinct in WT versus Nlrp6^-/-^ (**Figure S7**), suggesting possible connections linking CS, gut microbiota, and NLRP6-mediated airway inflammation. In line with this hypothesis, Nlrp6^-/-^ phenotype was characterized by decreased cell recruitment (**Figure 7F**), and, in particular, neutrophil influx (**Figure 7G**), BALF CXCL1 (**Figure 7H**), and CXCL5 (**Figure 7I**) levels were transferred to WT mice upon co-housing. These results indicate that the gut microbiota influences CS-induced airway inflammation. It is noteworthy that total cells (**Figure 7F**), especially neutrophils influx (**Figure 7G**), CXCL1 (**Figure 7H**), and CXCL5 (**Figure 7I**) secretions into the BALF, remained low in Nlrp6^-/-^ co-housed with WT mice (Nlrp6 CH), indicating that microbiota from Nlrp6^-/-^ mice display a dominant effect. These results highlight differences in gut microbiota composition between WT and Nlrp6^-/-^ mice and show that gut microbiota from Nlrp6^-/-^ mice limit airway inflammation following CS exposure.

### Oral transplantation of WT germ-free mice with fecal microbiota from Nlrp6^-/-^ mice attenuated CS exposure-induced lung inflammation

**Figure 8:**
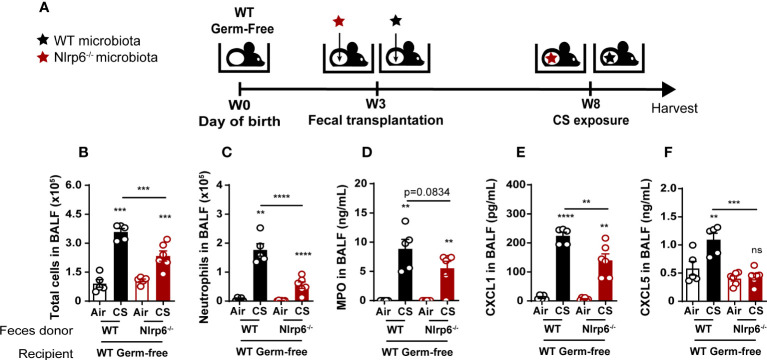
Attenuation of CS-induced lung inflammation in WT germ-free mice after oral transplantation with fecal microbiota from Nlrp6-/- mice. **(A)** WT germ-free (GF) mice were colonized with WT or Nlrp6-/- microbiota at weaning and let 5 weeks for microbiota implantation for before CS exposure. **(B)** BALF total cell and **(C)** neutrophil counts. **(D-F)** BALF MPO, CXCL1 and CXCL5 levels. Data are representative of two experiments and are expressed as mean values ± SEM (n= 4-6 mice per group), ns, non-significant, **p < 0.01, ***p < 0.001, ****P<0.0001.

To confirm that gut microbiota regulate airway inflammation from CS exposure in an NLRP6-dependent manner, we colonized germ-free (GF) WT mice (WT^GF^) with fecal microbiota obtained from WT or Nlrp6^-/-^ mice bred in the same EOPS mouse facility. Transplanted mice were maintained in isolators for 4 weeks to allow microbiota reconstitution and immune system maturation. Following one week of acclimation in a conventional breeding facility, mice were exposed to CS for 4 days (**Figure 8A**). While GF mice were unable to mount proper inflammatory response (43), we observed that reconstitution of WT^GF^ mice with WT microbiota (WT>WT^GF^) allows a normal lung inflammatory response to CS with CXCL1 and CXCL5 secretion in the bronchoalveolar space (**Figures 8B–F**). In contrast, WT^GF^ mice colonized with microbiota from Nlrp6^-/-^ mice (Nlrp6^-/-^>WT^GF^) displayed decreased cellular influx in BALF (**Figure 8B**), in particular neutrophil (**Figure 8C**) associated with decreased MPO (**Figure 8D**), CXCL1 (**Figure 8E**), and CXCL5 (**Figure 8F**) levels in BALF compared to WT>WT^GF^ mice. These results confirm that gut microbiota shape lung inflammation and that gut microbiota from Nlrp6^-/-^ mice negatively regulate lung inflammation upon acute CS exposure.

## Discussion

NOD-like receptor family pyrin domain containing (NLRP)6 inflammasome was shown to regulate microbiota homeostasis as well as antibacterial and antiviral immunity in the intestine (24–28, 41). However, the role of this multifaceted immune sensor in the lungs remains poorly understood. Studies showed that NLRP6 controls neutrophil recruitment and function during pulmonary bacterial infection (29, 30, 46–48), but its involvement in the context of sterile lung injury remains elusive. We chose to investigate early inflammation mechanisms because acute inflammation repetition often lead to chronicity and lung pathology. In addition, episodes of acute inflammatory exacerbations due to bacterial infections, frequently observed in COPD patients, are associated with dramatic increased pathology severity. Therefore, a better characterization of the mechanisms of acute phases of inflammation appears crucial.

We demonstrated that NLRP6 positively controls both pulmonary inflammation and remodeling following CS exposure. Nlrp6-deficient mice (Nlrp6^-/-^) displayed a drastic reduction of airway cell recruitment, particularly neutrophils, macrophages, and lymphocytes, and attenuated lung tissue inflammation in comparison to WT mice. Interestingly, NLRP6 contribution is likely context-dependent, as it was shown to negatively regulate pulmonary host defense after gram-positive bacterial infection through neutrophil influx modulation (29). COPD patients often suffer from disease exacerbations due to bacterial infections, and despite strong neutrophil-driven inflammation, these cells are largely ineffective, leading to impaired bacterial clearance (49–51). A possibility is that NLRP6 favors chronic detrimental neutrophilic inflammation but suppresses anti-bacterial immunity. If this is the case, targeting NLRP6 in COPD patients might be beneficial by preventing chronic inflammation and bacterial infection-driven disease exacerbation. This hypothesis could be tested in COPD exacerbation models by infecting chronically CS-exposed mice to bacteria such as Streptococcus pneumonia or Haemophilus influenza.

Our data demonstrate for the first time that NLRP6 is a sensor of lung injury, here induced by CS but possibly by other chemicals and toxics, opening new investigation fields. We verified that mouse genetic background and/or housing were not responsible for the difference in inflammatory response to CS observed using Nlrp6^+/+^ and Nlrp6^-/-^ littermate mice and observed similar decreased pulmonary inflammation in Nlrp6^-/-^ mice in comparison to Nlrp6^+/+^ littermates.

We provide evidence that NLRP6 expression in radioresistant cells positively regulates CS-mediated airway neutrophil inflammation. Our results show that NLRP6 is expressed in tracheal and lung epithelial cells and indicate that acute CS-exposure enhances NLRP6 expression in airway epithelial cells in mice. Although much less than in the intestine, NLRP6 was shown expressed in the lungs in epithelial cells and tissue-infiltrating neutrophils and macrophages after bacterial infection of humans and mice (29, 30, 46–48)).

Then, using mice deficient for Nlrp6 specifically in aquaporin-expressing lung cells (Nlrp6^fl/fl^ Acid^+/CRE^) and their control littermates (Nlrp6^fl/fl^ Acid^+/+^), we demonstrated that NLRP6 expressed in the lung controls neutrophilic airway and lung inflammation in response to acute CS exposure.

We confirmed that CXCL5, a neutrophilic chemokine essentially produced by airway epithelial cells, is a crucial chemokine in CS-induced pulmonary inflammation (11, 13, 39, 52, 53). In addition, exogenous recombinant CXCL5 was sufficient to restore neutrophilic inflammation in Nlrp6^-/-^ mice, suggesting that NLRP6 expressed in airway epithelial cells plays a key role in CXCL5 production and secretion and leads to neutrophil recruitment and airway inflammation.

We report a new role for the NLRP6 inflammasome in pulmonary inflammation from CS exposure through production of the neutrophilic chemokine CXCL1/KC and CXCL5/LIX, leading to neutrophil influx and airway inflammation. NLRP6 expression and function in airway epithelial cells highlight active contribution of tissue constitutive cells in pulmonary inflammation as reported recently (16, 17). In addition, the NLRP6, ASC, Casp-1, and/or Casp-11 inflammasome-related proteins contribute to CS-induced pulmonary inflammation. CS-exposure induced NLRP6 expression in AEC, suggesting the formation of the NLRP6 inflammasome in these cells. We recently reported that CS exposure induces NLRP3-dependent caspase-1 activation in bronchoalveolar space macrophages and NLRP3-dependent gasdermin D activation in both bronchoalveolar space macrophages and bronchial epithelial cells (54). This indicates that both NLRP3 and NLRP6 inflammasomes are involved in CS-induced pulmonary inflammation. However, the inflammasome-independent role of NLRP6 was reported in intestinal epithelial cells (28). Indeed, NLRP6 was showed to control enteric virus infection through its binding to viral RNA via the RNA helicase Dhx15 and interacted with mitochondrial antiviral signaling protein to induce type I/III interferons and IFN-stimulated genes (28). Even if we observed a similar reduction in inflammation in the absence of NLRP6, ASC, or Casp1/11, we cannot exclude the possibility that NLRP6 could play a role independently of inflammasome via unknown partners.

Here we hypothesized that gut microbiota from Nlrp6^-/-^ mice could influence pulmonary inflammation through a gut-to-lung axis. We showed that whole body bacterial depletion in WT mice impairs pulmonary inflammation upon CS exposure and recapitulates our results observed in Nlrp6^-/-^ mice. Interestingly, Nlrp6^-/-^ mice presented defective mucus secretion and intestinal barriers, reduced control of colonic host-microbial interface, and microbiota dysbiosis (25, 26). These results were challenged when Nlrp6^+/+^ and Nlrp6^-/-^ littermates were used, both displaying intact mucus layers (55). However, *ex vivo* LPS-induced mucus secretion was shown to be defective in intestinal goblet cells of colonic tissue from Nlrp6-/- mice in comparison to Nlrp6^+/+^ littermates (55).

Importantly, we report for the first time the existence of an NLRP6-dependent gut-to-lung axis controlling pulmonary inflammation following CS-induced injury. Co-housing and fecal transplantation experiments both revealed that a transfer of Nlrp6^-/-^ gut microbiota to WT mice impedes pulmonary inflammation in response to CS. During co-housing or fecal transplantation, metabolites generated by Nlrp6^-/-^ gut microbiota upon CS exposure may inhibit NLRP6 in airway epithelial cells of WT mice. In addition, antibiotic-mediated microbiota depletion indicates that the microbiome is required for efficient lung immunity. While antibiotic treatment probably affects both gut and lung microbiota, these data suggest that the predominant gut microbiota are necessary for CS-induced lung inflammation through NLRP6 activation and subsequent lung inflammation. Lung microbiota composition analysis is difficult to perform because of the low number of bacteria. However, we believe that as shown, NLRP6-dependent perturbation of the gut microbiome may generate specific metabolites or compounds (43, 56) that might modulate NLRP6 inflammasome activation in the lung.

Our study supports evidence of an altered gut microbiota between WT and Nlrp6^-/-^ mice as previously reported (25, 41, 43, 44). By analyzing gut microbiota phylogenetics after DSS-induced colitis of ASC-deficient mice, two studies suggested that the NLRP6 inflammasome does not shape commensal gut microbiota composition (57, 58). However, two other laboratories reported that littermate breeding led to the development of distinct microbiome compositions in NLRP6 inflammasome-deficient mice housed in two different facilities (27, 43).

In addition, two independent studies showed that NLRP6 deficiency led to impaired intestinal mucus secretion, disrupted mucus barrier, and mucosal surface invasion by enteric pathogens and finally to dysbiosis (25, 26), but this was discussed by another study (55). These seemingly conflicting results on the role of NLRP6 inflammasome might be explained by differences in microbiome of mouse lines used in different laboratories. Indeed, familial transmission was shown to significantly influence microbiota composition in conventionally housed Nlrp6^-/-^ mice. In addition, introduction of potential pathobionts revealed effects of Nlrp6 deficiency on gut microbiome, and increased abundance of pathobionts was observed in Nlrp6^-/-^ mice, indicating that Nlrp6^-/-^ microbiota depend on community structure (42). Moreover, the littermate approach has been coupled with the generation of germ-free mice. *De novo* dysbiosis was observed in spontaneous recolonization of germ-free NLRP6^-/-^ mice (43) as well as after fecal transfer of diverse microbiomes in germ-free NLRP6^-/-^ mice compared to germ-free WT mice (27, 42, 43).

Our results obtained after co-housing, fecal transplantation experiments, and antibiotics treatments demonstrate that airway exposure to CS significantly alters gut microbiota composition and hence may influence gut homeostasis and/or inflammation. These observations are in line with data showing the increased risk of smokers and COPD patients of developing intestinal diseases and support the notion of a complex interplay between environmental factors, gut microbiota, and organ disease (59, 60). Epidemiological evidence has linked gut dysbiosis and COPD (61, 62). Interestingly, some specific changes we observed in gut microbiota of CS-exposed Nlrp6^-/-^ mice are inversely correlated to fecal microbiome taxonomic indicators described in COPD patients. For example, upon CS exposure, Clostridia are increased in the gut microbiota of Nlrp6^-/-^ mice in comparison to WT mice (**Figure S5**) but depleted in the fecal microbiome of COPD patients in comparison to healthy subjects. Inversely, Bifidobacteriaceae are decreased in gut microbiota of Nlrp6^-/-^ mice whereas increased in the fecal microbiome of COPD patients (63).

In addition, severe COPD was associated with reduced sputum microbiota diversity (64) and lung microbiome dynamics during stability and exacerbation, which influences COPD pathogenicity (65, 66). It is likely that the lung microbiota may be influenced by CS exposure in our mouse model as shown in COPD patients (66). In particular, during infection-triggered acute COPD exacerbations, pathogens promote lung microbiota dysbiosis that may favor intestinal diseases, pointing to the existence of a bidirectional connection between the lungs and the gut in COPD (67). Altered lung microbiota in Nlrp6^-/-^ mice could interfere with host defense against lung infection, in particular triggered by Streptococcus pneumonia (*S.p*.) and frequently associated with COPD exacerbation (68). However, NLRP6 was shown to negatively regulate inflammation to *S.p* (31, 48) or to Staphylococcus aureus infections (29), suggesting a beneficial role of Nlrp6 depletion or inhibition in these secondary infections. However, NLRP6 might exhibit detrimental functions in other infection settings. We show that NLRP6 expression in both the lung and gut controls CS-induced pulmonary inflammation. Gut microbiota shaped by NLRP6 expressed in the gut regulates pulmonary inflammation from CS probably via circulating microbiota-derived metabolites (69). In addition to gut metabolites, lung inflammation can also be modulated by gut microbiota-derived components, such as lipoteichoic acid (LTA), a major constituent of the cell wall of gram-positive bacteria, which has been shown to induce inflammation through NLRP6 sensing in wild-type mice but not in Nlrp6^-/-^ mice (70). As a whole, we report new data supporting important NLRP6 functions in basal and pathological lung settings that could be translated to a clinical setting following preclinical validations. Gut microbiota-derived metabolites or components might represent biomarkers present in the blood of COPD patients (43, 71). In addition, this study uncovers NLRP6 as a new target for the development of potential therapeutic strategies against COPD by specific inhibition of NLRP6. In addition, gut microbiota transfer approaches might be useful in the treatment of COPD.

## Data availability statement

The original contributions presented in the study are publicly available. This data can be found here: https://trace.ncbi.nlm.nih.gov/Traces/?view=study&acc=SRP441823.

## Ethics statement

The animal study was approved by Ministère de l’enseignement supérieur, de la recherche et de l’innovation numéro APAFIS#26177-2019021818223038 v15. The study was conducted in accordance with the local legislation and institutional requirements.

## Author contributions

MN, MF, SH-M, MS, FS, NR, and AG performed the experiments. MN, NR, AG, PG, HS, and IC conceived the experiments and analyzed the data. MLB supervised mouse breeding. LA and MC provided mouse strains. JVS provided antibodies. MN, NR, BR, VQ, FT, PG, HS, and IC discussed the results. MN, NR, AG, HS, and IC wrote the manuscript. IC, FT, PG, and VQ provided funding, and IC provided overall supervision of this study. All authors contributed to the article and approved the submitted version.
