# Supplementary figures and images for: NLRP6 controls pulmonary inflammation from cigarette smoke in a gut microbiota-dependent manner
Source: Front Immunol. 2023 Dec 11;14:1224383. doi: 10.3389/fimmu.2023.1224383 (PMC10749332; doi:10.3389/fimmu.2023.1224383)

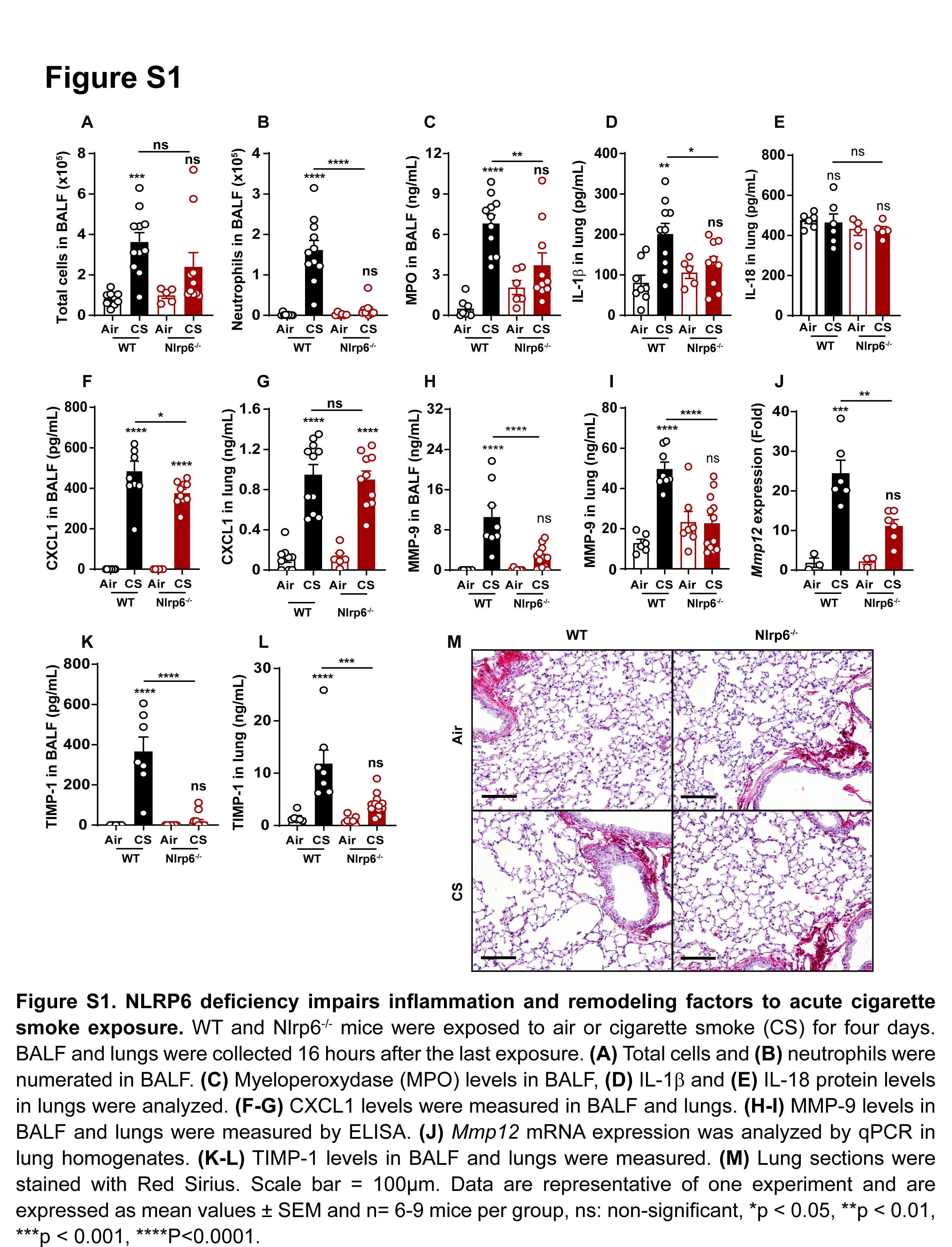

Supplement: Supplementary file 1 [file Image_1.tif]

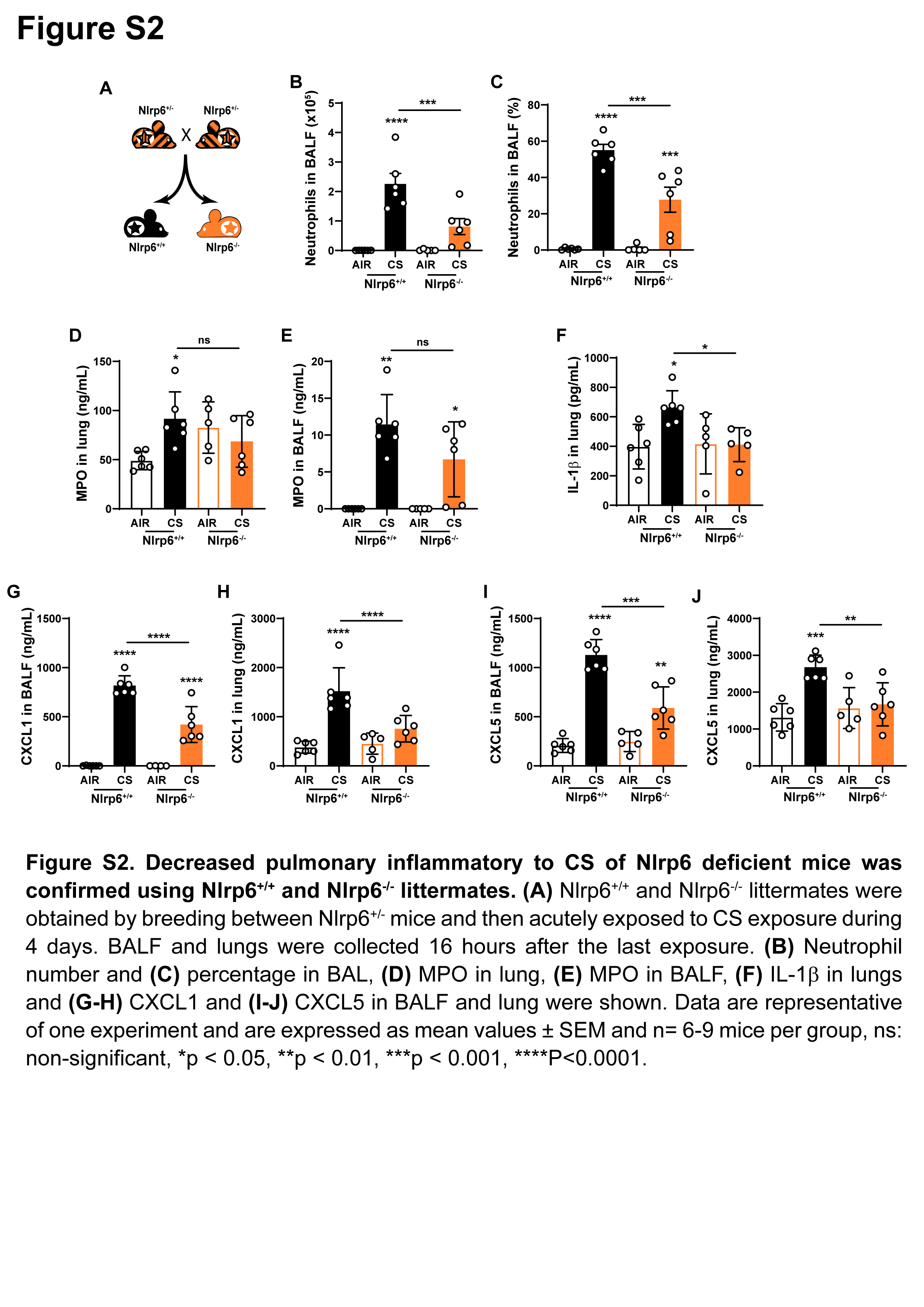

Supplement: Supplementary file 2 [file Image_2.tif]

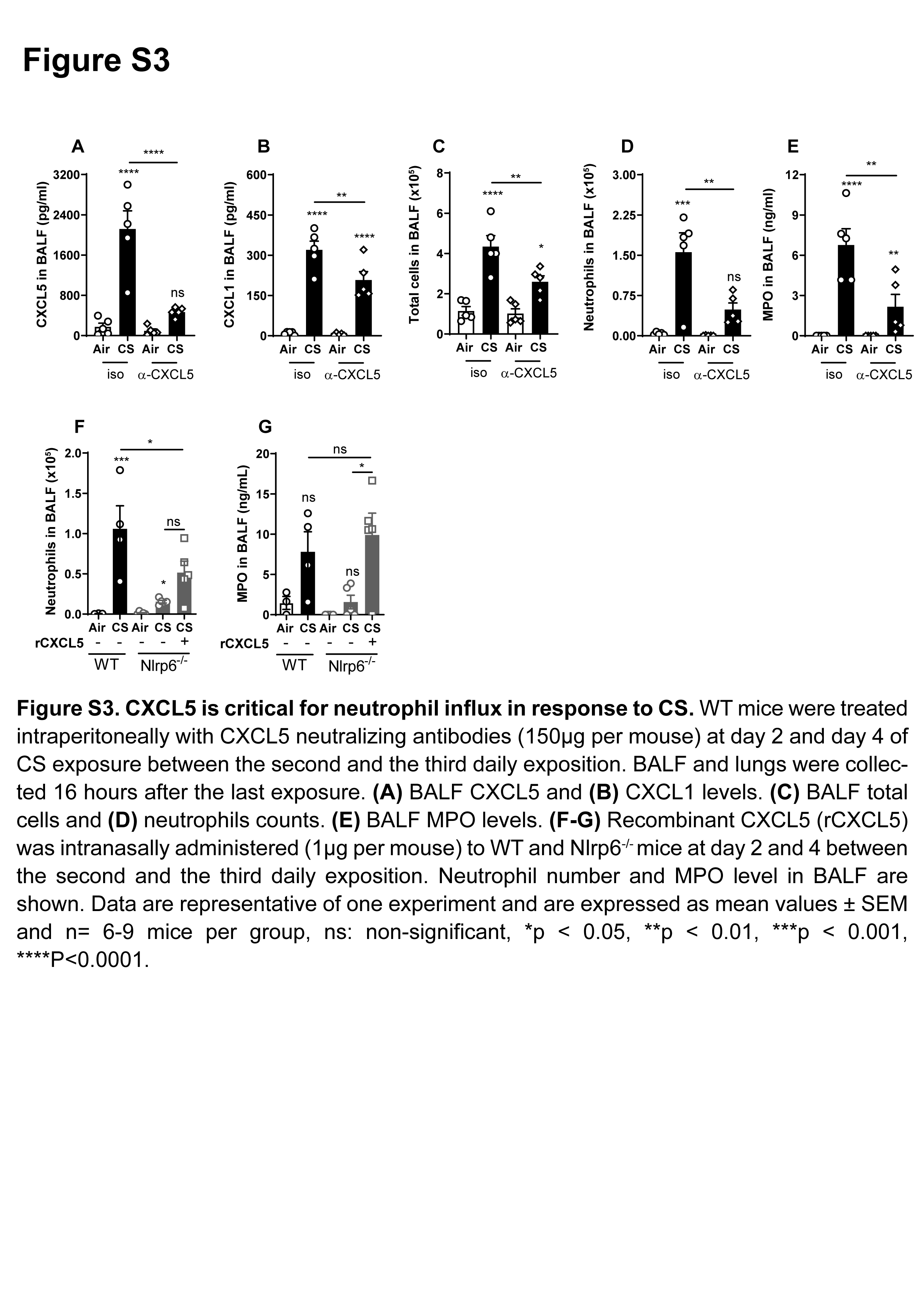

Supplement: Supplementary file 3 [file Image_3.tif]

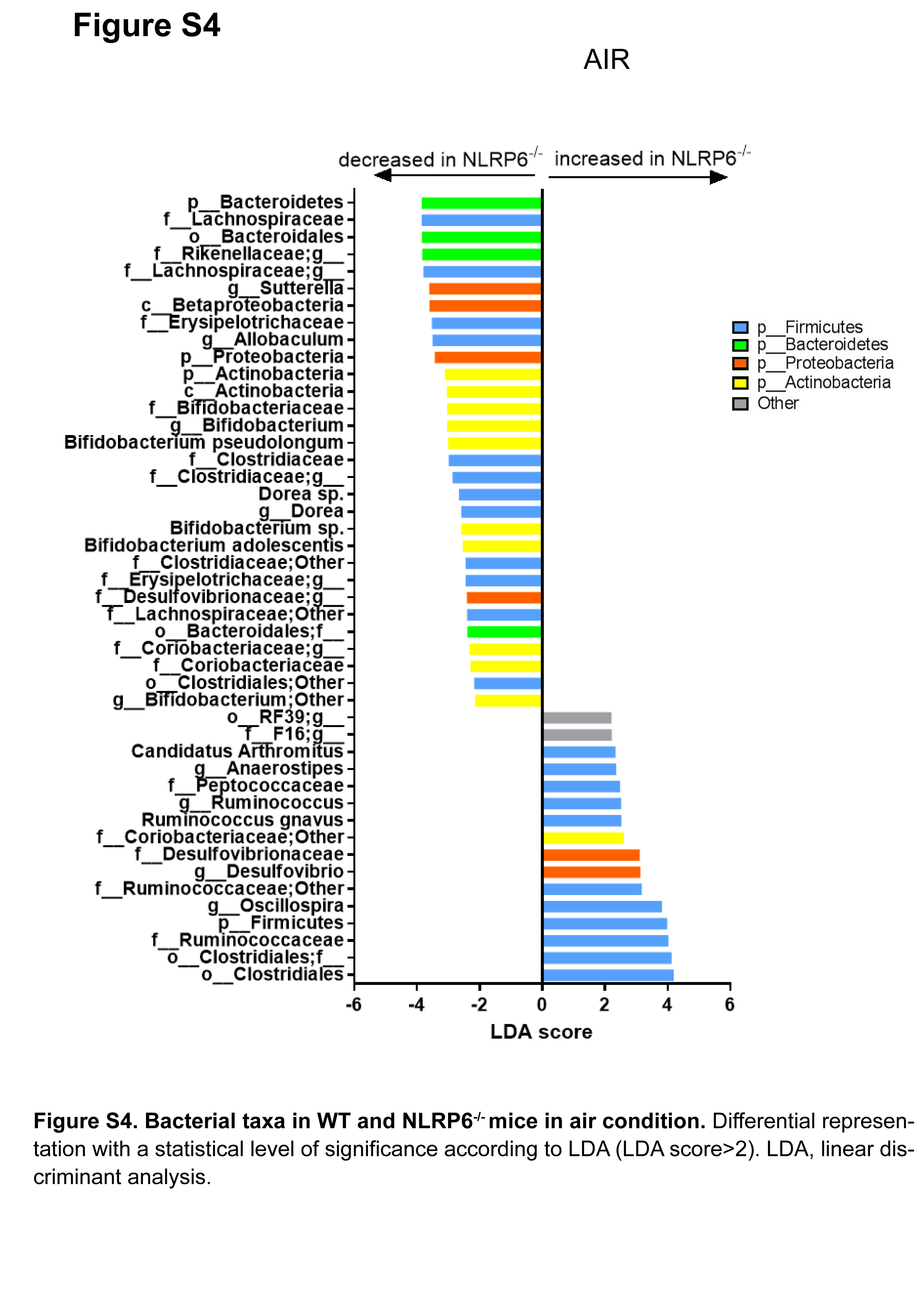

Supplement: Supplementary file 4 [file Image_4.tif]

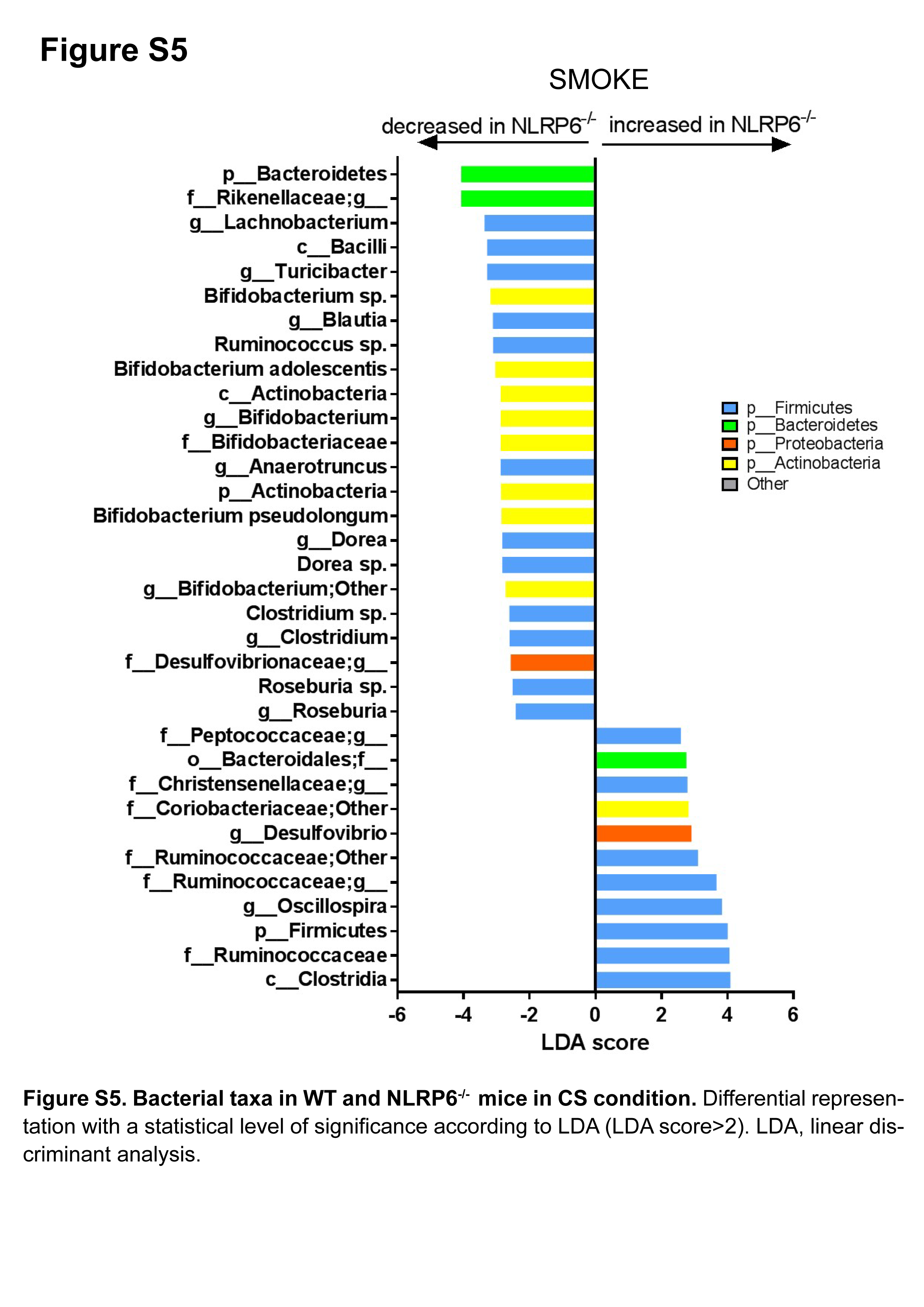

Supplement: Supplementary file 5 [file Image_5.tif]

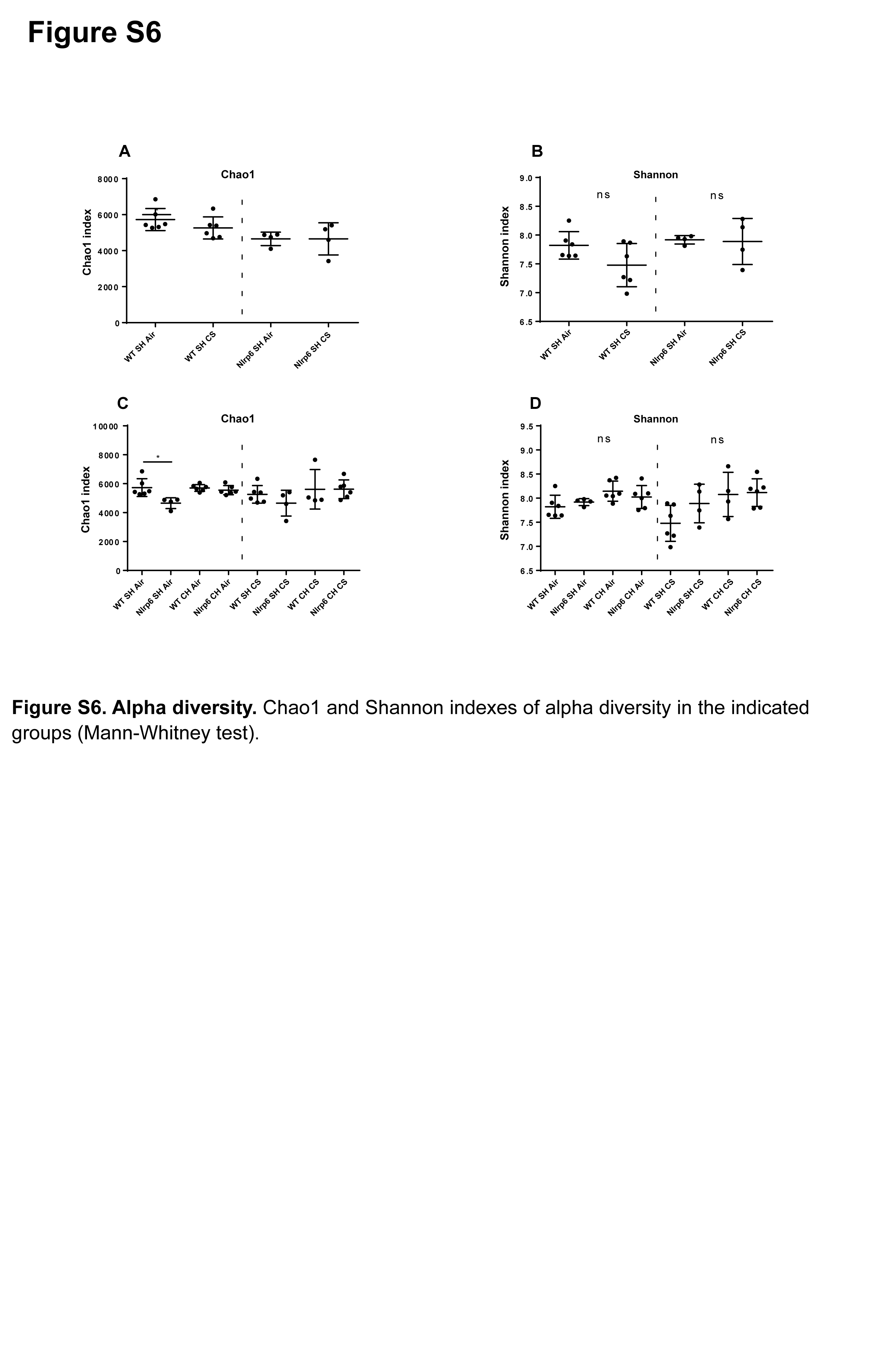

Supplement: Supplementary file 6 [file Image_6.tif]

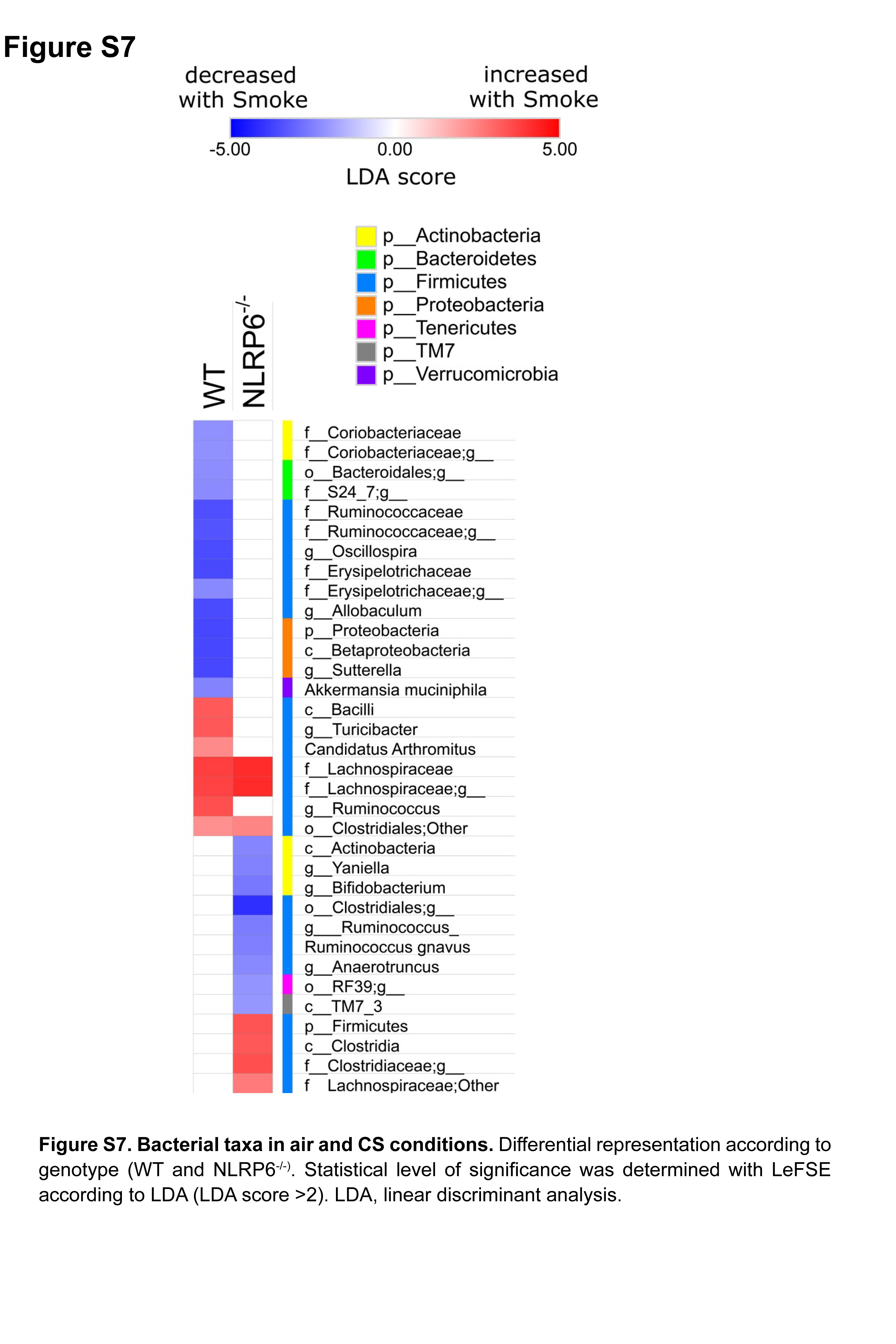

Supplement: Supplementary file 7 [file Image_7.tif]

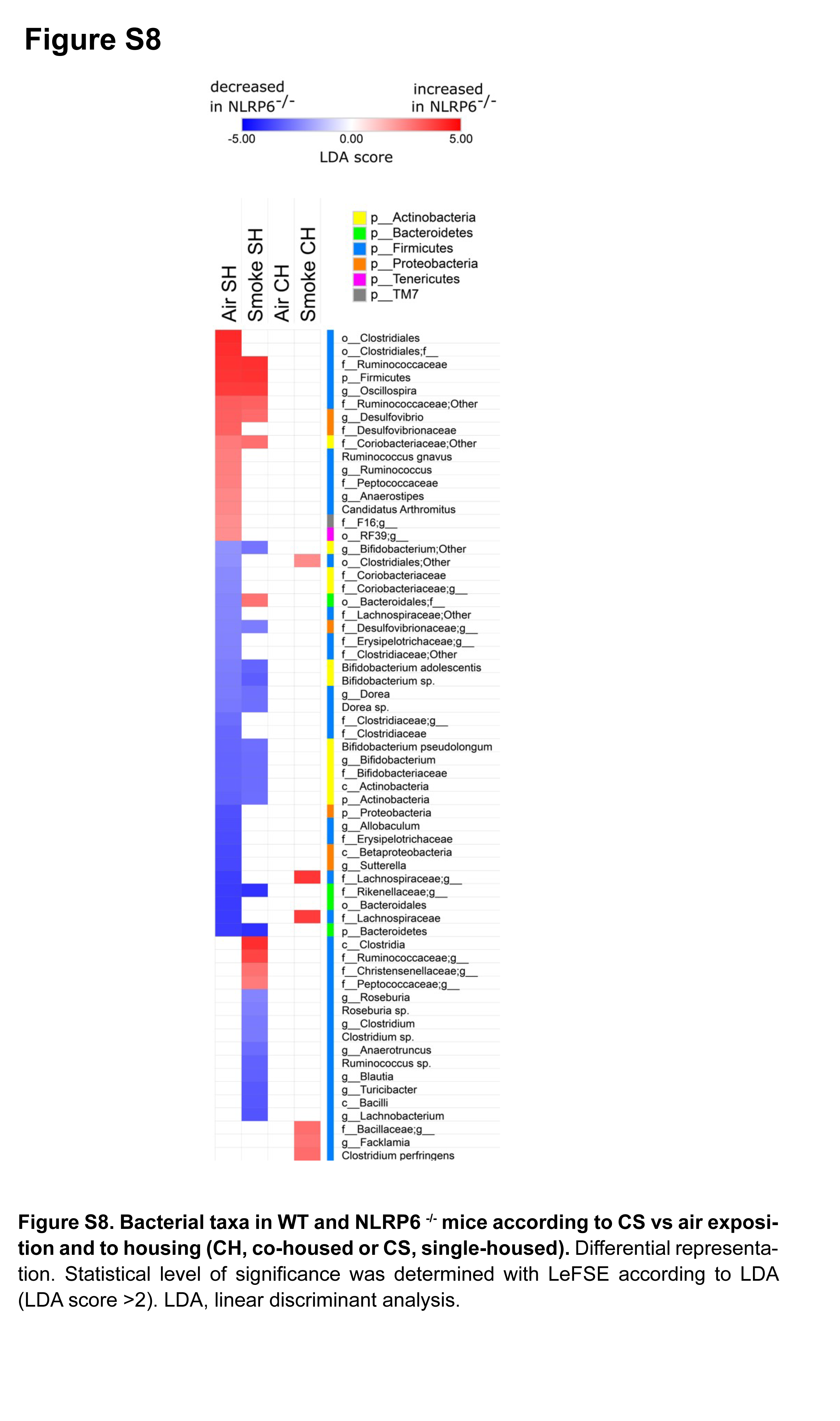

Supplement: Supplementary file 8 [file Image_8.tif]
